# Integrative analysis of the transcriptome and metabolome reveals the importance of hepatokine FGF21 in liver aging

**DOI:** 10.1016/j.gendis.2023.101161

**Published:** 2023-11-07

**Authors:** Wenchao Wang, Junjie Qian, Mingge Shang, Yiting Qiao, Jiacheng Huang, Xinxin Gao, Zhou Ye, Xinyu Tong, Kangdi Xu, Xiang Li, Zhengtao Liu, Lin Zhou, Shusen Zheng

**Affiliations:** a Division of Hepatobiliary and Pancreatic Surgery, Department of Surgery, First Affiliated Hospital, School of Medicine, Zhejiang University, Hangzhou, Zhejiang 310003, China; b NHC Key Laboratory of Combined Multi-organ Transplantation, Hangzhou, Zhejiang 310003, China; c Key Laboratory of the Diagnosis and Treatment of Organ Transplantation, Research Unit of Collaborative Diagnosis and Treatment for Hepatobiliary and Pancreatic Cancer, Chinese Academy of Medical Sciences (2019RU019), Hangzhou, Zhejiang 310003, China; d Key Laboratory of Organ Transplantation, Research Center for Diagnosis and Treatment of Hepatobiliary Diseases, Hangzhou, Zhejiang 310003, China; e Shulan International Medical College, Zhejiang Shuren University, Hangzhou, Zhejiang 310015, China; f Shulan (Hangzhou) Hospital Affiliated to Zhejiang Shuren University Shulan International Medical College, Hangzhou, Zhejiang 310000, China

**Keywords:** Antioxidant capability, FGF21, Liver aging, Liver regeneration, Multiomics analysis

## Abstract

Aging is a contributor to liver disease. Hence, the concept of liver aging has become prominent and has attracted considerable interest, but its underlying mechanism remains poorly understood. In our study, the internal mechanism of liver aging was explored via multi-omics analysis and molecular experiments to support future targeted therapy. An aged rat liver model was established with d-galactose, and two other senescent hepatocyte models were established by treating HepG2 cells with d-galactose and H_2_O_2_. We then performed transcriptomic and metabolomic assays of the aged liver model and transcriptome analyses of the senescent hepatocyte models. In livers, genes related to peroxisomes, fatty acid elongation, and fatty acid degradation exhibited down-regulated expression with aging, and the hepatokine Fgf21 expression was positively correlated with the down-regulation of these genes. In senescent hepatocytes, similar to the results found in aged livers, FGF21 expression was also decreased. Moreover, the expressions of cell cycle-related genes were significantly down-regulated, and the down-regulated gene E2F8 was the key cell cycle-regulating transcription factor. We then validated that FGF21 overexpression can protect against liver aging and that FGF21 can attenuate the declines in the antioxidant and regenerative capacities in the aging liver. We successfully validated the results from cellular and animal experiments using human liver and blood samples. Our study indicated that FGF21 is an important target for inhibiting liver aging and suggested that pharmacological prevention of the reduction in FGF21 expression due to aging may be used to treat liver aging-related diseases.

## Introduction

The liver is an important organ, and its metabolic functions, such as energy and nutrient metabolism, are vital for life, which indicates that this organ is critical to human health. However, the risk of liver diseases increases significantly with aging, and the poor prognosis and severity of hepatic diseases are associated with advanced aging.^1^ The senescence of hepatocytes activates stellate cells in the liver, which promotes the progression of fibrosis.^2^ The aged liver exhibits a decreased regeneration ability and experiences oxidative stress; thus, ischemia-reperfusion further damages aged grafts during liver transplantation.^3^ However, due to the growing number of patients waiting for liver transplantation, the use of marginal grafts has also been maximized to expand the donor pool.^4^ Thus, more livers from the older are being used in liver transplantation. However, advanced donor age is a contributor to poor liver quality, and thus, high incidences of graft loss and complications, including thrombosis of the hepatic artery and ischemic-type biliary complications, are observed following liver transplantation from older donors.^5^ In addition, senescent hepatocytes can be regarded as storage pools for damaged cells; once they re-enter the cell cycle, there is a risk of cancer-causing mutations. Moreover, senescent cells secrete proinflammatory factors and matrix degradation molecules, which can stimulate cancer cell proliferation and epithelial–mesenchymal transition.^2^ Additionally, the function of senescent hepatocytes decreases and decompensates, which may result in insulin resistance or type-2 diabetes mellitus.^2^^,^^6^, ^7^, ^8^ Hence, scientists need to study the underlying mechanism of liver aging to discover future therapeutic targets.

At present, anti-aging is a promising and challenging field of study due to the complexity of aging mechanisms. Many studies on lifespan extension have been performed, and these include studies involving dietary restriction, exercise, and clearing of senescent cells.^8^^,^^9^ Among these strategies, dietary restriction has been found to enhance lipid degradation and autophagy by histone demethylase of JMJD3 to improve liver metabolism and extend the lifespan.^10^ Hill CM et al found that dietary protein restriction can improve liver metabolism and extend the lifespan.^11^ Moreover, lipophagy is promoted by exercise and dietary intervention to attenuate nonalcoholic fatty liver disease and liver aging.^12^ However, the underlying mechanism of hepatic aging remains poorly understood, and the studies conducted thus far on liver aging have lacked a systematic medical perspective, which can be obtained by multi-omics analysis. Therefore, further mechanistic investigation involving multiomics analysis is needed.

The analysis of omics data from livers offers potential ways to paint a panoramic picture of the environment in which the liver works in the body. The molecular functions and mechanisms in complex phenotypes can be unveiled by novel advanced high-throughput omics approaches, which are based on the metabolome, proteome, and genome.^13^ Techniques that integrate the data from transcriptomics and metabolomics can offer convincing evidence, and mutual validation of this evidence would allow the construction of reliable networks for unveiling the biological processes ranging from genes to the final metabolites.^14^ We can construct networks using omics data that integrate clinical information from individual patients to boost diagnostic efficiency or prognostic predictions of diseases.^14^^,^^15^ This study aimed to apply multi-omics methods to study the potential mechanism responsible for liver aging and thus identify key therapeutic targets. The results were then verified through molecular experiments.

Due to cost and time limitations, mimetic aging models are widely preferable to natural liver aging for research on the underlying mechanisms of liver aging.^16^ In many studies, d-galactose (D-gal) and H_2_O_2_ were used to induce oxidative stress *in vivo* and *in vitro* to mimic the natural process of liver aging in cell lines and rats.^16^, ^17^, ^18^, ^19^, ^20^, ^21^, ^22^, ^23^, ^24^, ^25^, ^26^ Increasing oxidative damage is an important contributor to aging. D-gal and H_2_O_2_ are both contributors to damage from oxidative stress, and galactose oxidase can oxidize D-gal into aldehydes and H_2_O_2_.^17^ To mimic hepatic aging, HepG2 cells have been widely used to construct senescent hepatocyte models.^24^^,^^27^, ^28^, ^29^ In addition, HepG2 cells have been used to study the lipid metabolism of hepatocytes.^30^, ^31^, ^32^ Zhao H et al studied remote liver injury correlated with ischemia-reperfusion injury of renal allografts in rats using HepG2 cells to mimic hepatocytes.^33^ In this study, to mimic natural hepatocyte aging, HepG2 cells were treated with H_2_O_2_ and D-gal, and thus two types of hepatocyte senescence models were established. In addition, a model of aged livers was constructed with D-gal. We then performed multi-omics analyses of these models to explore the internal mechanism of liver aging to support future targeted therapy. The expression of genes correlated with peroxisomes, fatty acid elongation, and degradation was significantly down-regulated in the aged liver model, and these genes were positively related to the hepatokine Fgf21. Furthermore, we found that FGF21 expression was also decreased in the models of senescent hepatocytes. In addition, in the senescent hepatocyte models, the cell cycle was also down-regulated, and the down-regulated gene E2F8 was the key cell cycle-related transcription factor (TF). Overexpression of FGF21 can protect against liver aging and inhibit the declines in the antioxidant and regenerative capacities associated with liver aging. Finally, we successfully validated our results from cellular and animal experiments using human liver and blood samples.

## Methods

### Study design

**Figure S1:**
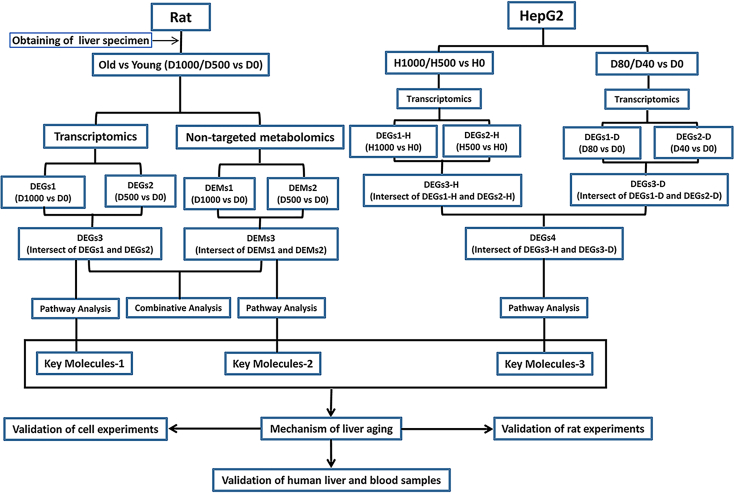
Flow diagram of the study design. DEGs, differentially expressed genes; DEMs, differentially expressed metabolites; DEGs-H, differentially expressed genes-H_2_O_2_; DEMs-D, differentially expressed metabolites-D-gal.

This research flowchart is illustrated in Figure S1. Aging was induced in the two groups (D1000 and D500) of rats with D-gal (at 500 mg/kg per day and 1000 mg/kg per day, respectively). Metabolomic and transcriptomic data were obtained from the livers of aged and young rats. The collected datasets included differentially expressed genes 1 (DEGs1), which contained the DEGs identified from a comparison of the D1000 and D0 groups; differentially expressed genes 2 (DEGs2), which contained the DEGs identified from a comparison of the D500 and D0 groups; and differentially expressed genes 3 (DEGs3), which contained the genes found in both DEGs1 and DEGs2. Similarly, nontargeted metabolomics yielded the differentially expressed metabolites 3 (DEMs3) dataset. An integrative analysis of DEGs3 and DEMs3 was performed. Additionally, pathway analyses of DEGs3 and DEMs3 were performed to identify the key molecule-1 and key molecule-2 datasets, respectively. We established hepatocyte senescence models with D-gal and H_2_O_2_. Two groups of aged hepatocytes, H1000 and H500, were established with 1000 μmol/L and 500 μmol/L H_2_O_2_, respectively. Transcriptomic information of aged and young hepatocytes was obtained. The DEGs1-H_2_O_2_ (DEGs1-H) dataset contained the DEGs identified from a comparison of the H1000 and H0 groups; the DEGs2-H_2_O_2_ (DEGs2-H) dataset contained the DEGs identified from a comparison of the H500 and H0 groups; and the DEGs3-H_2_O_2_ (DEGs3-H) dataset contained the DEGs found in both DEGs1-H and DEGs2-H. Additionally, two groups of aged hepatocytes, the D80 and D40 groups, were treated with 80 mg/mL and 40 mg/mL D-gal, respectively. In the above-described analysis, we obtained the DEGs3-D-gal (DEGs3-D) dataset. We then obtained the DEGs4 dataset from the intersection of the DEGs3-D and DEGs3-H datasets. A pathway analysis of DEGs4 was performed to obtain the key molecule-3 dataset. An interactive analysis of the key molecule-1, key molecule-2, and key molecule-3 datasets revealed the key mechanism of liver aging, and the elucidated mechanism was validated through cellular and animal experiments. In addition, we successfully validated the results from the cellular and animal experiments using human liver and blood samples.

### Animal experiments

Adult male Sprague Dawley rats lived in a 12-h/12-h light/dark cycle in the animal center for the experiment. The rats were given D-gal (500 mg/kg per day or 1000 mg/kg per day) for 8 weeks to induce aging, and the rats in the control group were injected with a normal saline solution. Additionally, for liver-specific up-regulation of Fgf21, the rats in the experimental group were injected with HBAAV-TBG-Fgf21-Flag-ZsGreen (1 × 10^12^ vg/mL), and the control rats were injected with HBAAV-TBG–NC–ZsGreen (1 × 10^12^ vg/mL). The liver malondialdehyde (MDA) levels were measured using the MDA assay kits (Beyotime, S0131 and S0101). Additionally, the biochemistry of the liver was measured with a Mindray BS-220. All animal experiments were approved by the Animal Committee for Ethics of the First Affiliated Hospital, College of Medicine, Zhejiang University (No. 2023957).

### Cellular experiments

HepG2 cells were cultured in Dulbecco's modified Eagle's medium (L111-500; Tecono) containing 10% fetal bovine serum (F8687, Sigma–Aldrich). A humidified incubator at 37 °C with 5% CO_2_ was used for cell culture. Aging was induced in HepG2 cells through treatment with D-gal (80 mg/mL or 40 mg/mL) and H_2_O_2_ (1000 μM or 500 μM) for 48 h, and the cells were then used for transcriptomic and metabolomic assays. Additionally, FGF21 was overexpressed by plasmid transfection in HepG2 cells.

### Human sample collection

A total of 12 healthy liver tissues (6 young and 6 elderly) were collected from the donor livers for transplantation. Liver samples from donors older than 60 years of age comprised the elderly group. The mean age (± SD) of the elderly group was 71.5 ± 6.02 years, and that of the young group was 31.5 ± 2.07 years. The liver samples were flash-frozen with liquid nitrogen and stored in a biobank (−80 °C). These liver samples were used for Western blotting (WB) analysis.

Moreover, 120 samples of blood (60 young and 60 elderly) were obtained from the donors for liver transplantation. Donors older than 60 years of age were also regarded as the elderly group. The mean age (± SD) of the elderly group was 65.22 ± 4.40 years, and that of the young group was 36.97 ± 5.77 years. These blood samples were used for enzyme-linked immunosorbent assays (ELISAs).

A total of 100 healthy liver samples (50 young and 50 elderly) were collected from the donor livers for transplantation. Liver specimens from donors older than 60 years of age were considered the elderly group. The mean age (± SD) of the elderly group was 67.42 ± 5.34 years, and that of the young group was 34.56 ± 7.89 years. These tissues were fixed in 4% paraformaldehyde and embedded in paraffin. These paraffin-embedded tissues were then used to prepare tissue microarrays and immunohistochemical staining was performed on these tissue microarrays.

The studies associated with human participants were reviewed and approved by the Shulan (Hangzhou) Hospital Affiliated to Zhejiang Shuren University, Shulan International Medical College (KY2021023). We obtained informed consent from each subject. Moreover, the experiments conformed to the principles of the Department of Health and Human Services Belmont Report and WMA Declaration of Helsinki.

### Quantitative real-time polymerase chain reaction (RT–qPCR)

For RNA isolation, we resolved cells or tissues in TRIzol reagent (15596026; Invitrogen). The RNeasy Mini Kit (74104; Qiagen) was used for the purification of RNA. We measured the expression of target genes by RT‒qPCR using SYBR Green Master Mix (1725121; Bio-Rad) and an integrative detection system (CFX96; Bio-Rad). Table S1 lists the primer sequences. The delta–delta Ct method was used for the comparison of gene expression,^34^ and GAPDH was used as an internal reference.

### WB analysis

RIPA lysis buffer (P0013, Beyotime) was used for the lysis of human and rat tissues and cells for protein extraction. Anti-Fgf21 (A3908), anti-Creb3l3 (A16655), anti-E2F8 (A1135), anti-superoxide dismutase 1 (SOD1) (A0274), and anti-Flag (AE005) antibodies were purchased from ABclonal (Wuhan, China). Anti-P16 (10883-1-AP), anti-P21 (28248-1-AP), anti-P53 (10442-1-AP), anti-cyclin A2 (66391-1-lg), anti-cyclin D1 (60186-1-AP), anti-Cyclin E1 (11554-1-AP), anti-catalase (66765-1-lg), and anti-cyclin B1 (67686-1-lg) antibodies were purchased from Proteintech (Chicago, IL, USA). Anti-MMP-3 (#56062), anti-HMGB1 (#56062), anti-Lamin B1 (#56062), and anti-P53 (#56062) antibodies were purchased from Cell Signaling Technology (Beverly, MA, USA). Anti-P16 (sc-1661) antibody was purchased from Santa Cruz (Santa Cruz, CA, USA). Anti-Fgf21 (ab171941) and anti-Flag (ab205606) antibodies were purchased from Abcam (Cambridge, UK). A BCA Protein Assay Kit (FD2001, FDbio Science) was used for protein quantification, and we loaded and separated a total of 30 μg of protein on SDS‒PAGE gels. We transferred the isolated proteins to nitrocellulose membranes and blocked them in 5% nonfat milk for 1 h. For detection, the membranes were then incubated overnight with the primary antibodies. The immunoblots were washed a total of three times (10 min each) with tris-buffered saline solution containing 0.1% Tween 20 and incubated with horseradish peroxidase-conjugated secondary antibodies for 1 h. The blots were then washed three times (10 min each) with a tris-buffered saline solution containing 0.1% Tween 20, developed with an enhanced chemiluminescence kit for horseradish peroxidases (FD8020, FDbio Science), and visualized with a Quantity One System image analyzer (ChemiScope620014-8Q, Clinx Science Instruments).

### Immunohistochemical staining

For immunohistochemistry, tissue microarrays from paraffin-embedded liver samples were subjected to antigen retrieval by incubation in EDTA buffer (pH 9.0) in an induction cooker for 25 min. After treatment with goat serum at 37 °C for 40 min, the tissue sections were incubated at 4 °C overnight with the following antibodies: anti-CDKN2A/p16INK4 (ab270058), anti-p21 Waf1/Cip1(12D1) (#2947), anti-p53 (ab32389), anti-MMP3 (ab137659), anti-lamin B1 (ab229025), anti-FGF21 (ab171941), anti-CREB3L3 (ab150865), Anti-E2F8 (DF6269), anti-PCNA (ab92729), anti-SOD1 (ab51254), and anti-catalase (ab76024). Anti-p21 Waf1/Cip1(12D1) (#2947) was purchased from Cell Signaling Technology (Beverly, MA, USA), and the others were purchased from Abcam (Cambridge, UK). For immunohistochemistry, the slides were incubated with biotin-conjugated secondary antibodies (ZSGB-BIO). The immunohistochemical staining intensity was estimated based on score of relative expression (Table S2).^26^ The results of five random fields (HPF, × 400 magnification) were averaged and used for statistical analysis.

### Senescence-associated β-galactosidase (SA-β-Gal) staining

A senescence β-galactosidase staining kit (C0602, Beyotime) was used for SA-β-Gal staining. We used a Nikon Eclipse Ti inverted microscope to visualize the cells.

### RNA sequencing (RNA-seq)

RNA-seq was performed using HepG2 cells and liver tissues from rats. We used an Illumina NovaSeqTM 6000 sequence platform to sequence the cDNA libraries to produce a total of 2 × 150-bp paired-end reads. We examined one library of reads per biological sample for sequencing errors prior to mapping. We used the HISAT2 package (https://daehwankimlab.github.io/hisat2/, version: 2.0.4) for sequencing alignment. Further details are provided in the Supplementary Materials.

### Metabolomic analysis

Liver tissues were subjected to nontargeted metabolomic assays. Metabolic analysis was performed by liquid chromatography-mass spectrometry with an ultrahigh-performance liquid chromatograph coupled to a QE plus system (Thermo Fisher Scientific, USA) in both electrospray ionization-negative and positive modes.^35^ The original file (.raw) obtained after mass spectrometry detection was imported into Compound Discoverer 3.2 (Thermo Fisher Scientific, USA, version 3.2) software, spectrum processing, and database searching were conducted to obtain qualitative and quantitative results, and quality control of the data was then performed to ensure the accuracy and reliability of the data results. More details are provided in the Supplementary Materials.

### Principal component analysis (PCA) and orthogonal partial least-squares discriminant analysis (OPLS-DA)

PCA and OPLS-DA were conducted using OmicShare tools, a free online platform for data analysis (https://www.omicshare.com/tools), to evaluate the discrimination of transcriptomic and metabolomic profiles between the aged and non-aged groups, respectively. Further details are provided in the Supplementary Materials.

### Identification of TFs

We downloaded TFs from the AnimalTFDB3 website (hust.edu.cn) and analyzed the co-expression relationships between the TFs and DEGs by Pearson correlation analysis using OmicShare tools (https://www.omicshare.com/tools).

### Enrichment and pathway analyses, and network construction based on multi-omics datasets

DEM sets were analyzed by *t*-tests using the OmicShare tools (https://www.omicshare.com/tools), and the *P* value was adjusted for multiple tests based on the false discovery rate (Benjamini–Hochberg). The fold change is the ratio of the mean quantitative value of each metabolite of all biological replicates in the comparison group. We calculated the value of variable importance in the projection for each covariate by OPLS-DA using OmicShare tools (https://www.omicshare.com/tools), and variable importance in the projection > 1 indicated that the metabolites were important. The threshold of DEMs was set to variable importance in the projection > 1.0, log2-transformed expression fold change ≥ 0.26 or ≤ −0.32, and *P* value < 0.05. Based on the Kyoto Encyclopedia of Genes and Genomes (KEGG) database, potential metabolites were enriched for pathway imputation using the online toolkit "MetaboAnalyst" (https://www.metaboanalyst.ca/). Pathway significance was defined by *P* < 0.05. Further details regarding data processing are provided in the Supplementary Materials.

The DEG sets identified between two different groups using the OmicShare tools (https://www.omicshare.com/tools) were imputed into DESeq2 software (version: 1.40.2). Genes with log2-transformed expression fold change ≥ 0.26 or ≤ −0.32 were considered up-regulated DEGs or down-regulated DEGs, respectively, and all these DEGs had false discovery rate values below 0.05. The pathways potentially enriched in the significant DEGs were identified by KEGG analysis using the abovementioned OmicShare tools (https://www.omicshare.com/tools). Differential traits were identified by a joint pathway analysis of metabolic and transcriptomic databases, and the pathway maps integrating transcriptomic and metabolomic data were visualized using "Pathview" (https://pathview.uncc.edu/, version: 1.40.0).^36^ Pathway significance was defined by *P* < 0.05. Further data processing details are provided in the Supplementary Materials.

Transcriptional regulatory networks were then constructed based on connections among TFs, targeted genes, and metabolites in significantly enriched pathways. We calculated the Pearson correlation coefficient among TFs, genes, and metabolites using OmicShare tools (https://www.omicshare.com/tools). The network was visualized, and hub TFs, targeted genes, and metabolites were screened using Cytoscape (version: 3.9.1).^37^ Based on the TFs, targeted genes, and metabolites that were significantly correlated (*P* < 0.05), networks were constructed based on the metabolomic and transcriptomic analyses using the "cytoHubba" module (version: 0.1). Specifically, hub TFs, targeted genes, and metabolites were screened using appropriate algorithms in this module. We incorporated regulatory networks based on TFs, targeted genes, and metabolites that were associated with key traits in the prior pathway analysis. In addition, coexpressed protein–protein interaction networks were built based on DEGs located in significant pathways and their correlated TFs from the STRING database (https://cn.string-db.org/, version: 12.0).

We visualized the descriptive data and their interactive relationships by heatmaps, bar charts, Venn diagrams, and volcano, bubble, and scatter plots using OmicShare tools (https://www.omicshare.com/tools) to assess the discrimination of transcriptomic and metabolomic profiles.

### ELISA experiments

The FGF21 levels in the culture supernatant of HepG2 cells and human serum were determined by ELISA using a human FGF-21 ELISA kit (ELH-FGF21-1; Raybio, USA) according to the manufacturer's instructions.

### Cell cycle analysis

We seeded HepG2 cells in 6-well plates. After processing, these cells were collected and stained using a Cell Cycle Detection Kit (MultiSciences, China). The cell cycle ratios were detected by flow cytometry and quantified using ModFit (Version:5.0) software.

### Statistical comparisons

Nonnormally distributed data were log-transformed. The mean ± SD was used to describe the normally distributed data, and data were compared by one-way ANOVA. The median (interquartile range) was used to describe the nonnormally distributed data, and these data were compared by the nonparametric Mann–Whitney *U* test. The relationships among continuous, rank, and ordinal covariates were evaluated by correlation analysis using Pearson's, Spearman's, and Kendall's coefficients, respectively.

## Results

### Creation of the aged liver model and senescent hepatocyte models

**Figure 1:**
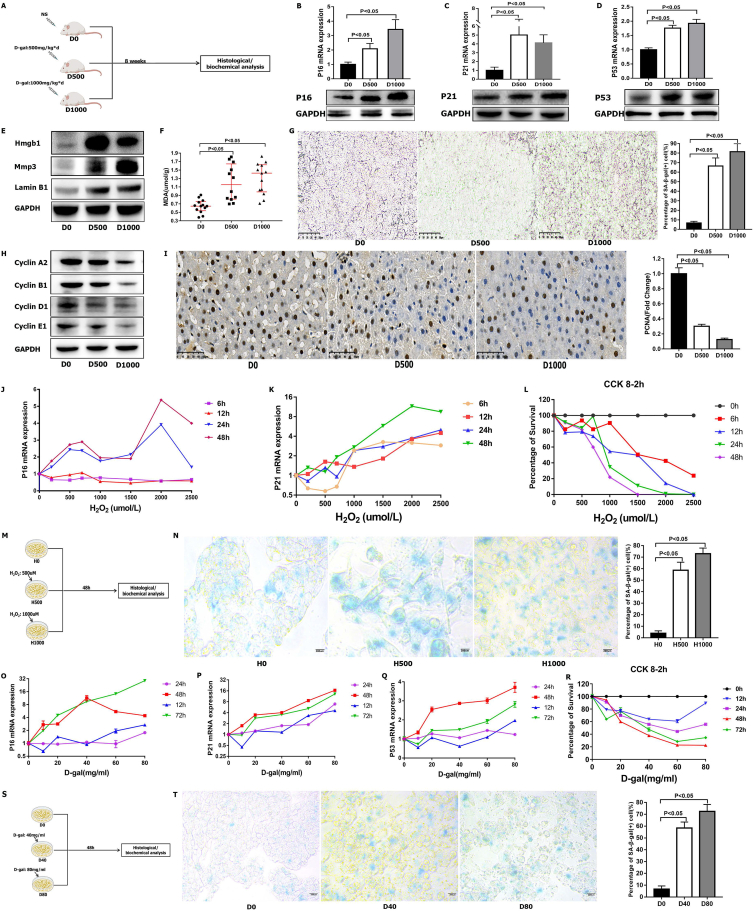
Induction of models for aged livers and senescent hepatocytes. **(A)** Induction of models for aged livers for further histological and biochemical analysis. **(B‒D)** RT‒qPCR for the expression levels of P16 (B), P21 (C), and P53 (D) in livers. The data were presented as mean ± standard error of the mean (SEM). The P16, P21, and P53 protein levels were validated by Western blotting (WB). **(E)** WB for the expression levels of Hmgb1, Mmp3, and Lamin B1 in livers. **(F)** The levels of MDA in livers. The data were presented as median with interquartile range. MDA, malondialdehyde. **(G)** SA-β-Gal staining in livers. The data were presented as mean ± SEM. **(H)** WB for the expression levels of Cyclin A2, Cyclin B1, Cyclin D1, and Cyclin E1 in livers. **(I)** Immunohistochemical staining for the expression of Pcna in livers. The data were presented as mean ± SEM. **(J, K)** RT‒qPCR for the expression levels of P16 (J) and P21 (K) in HepG2 cells. **(L)** CCK-8 experiments examining the percentage of cell survival after treatment with H_2_O_2_. **(M)** Induction of models for senescent hepatocytes by H_2_O_2_ for further histological and biochemical analysis. **(N)** SA-β-Gal staining in cells. The data were presented as mean ± SEM. **(O‒Q)** RT‒qPCR for the expression levels of P16 (O), P21 (P), and P53 (Q) in HepG2 cells. **(R)** CCK-8 experiments examining the percentage of cell survival after treatment with D-gal. **(S)** Induction of senescent hepatocyte models with D-gal for further histological and biochemical analyses. **(T)** SA-β-Gal staining in cells. The data were presented as mean ± SEM.

We selected a total of 24 male Sprague Dawley rats for the D0 (D-gal: 0 mg/kg per day), D500 (D-gal: 500 mg/kg per day), and D1000 (D-gal: 1000 mg/kg per day) groups. The aged liver model was induced by intraperitoneal injection of D-gal into rats for 8 weeks (D500 and D1000 groups) (Fig. 1A). D-gal was first used to induce aging in 1962 and has since been used widely.^16^ Advanced glycation end products are produced by the reaction of D-gal with free amines of amino acids and lead to the progression of liver diseases.^38^ In addition, galactose oxidase can oxidize D-gal into H_2_O_2_, which results in the formation of reactive oxygen species. Aldose reductase can also convert D-gal into galactitol, and galactitol cannot be further metabolized in cells. The increase in galactitol in the cells causes an increase in free radicals, which subsequently disrupts the normal osmotic pressure and redox imbalance. Hence, the formation of advanced glycation end products and reactive oxygen species, redox imbalance, and osmotic stress ultimately lead to aging in organisms.^16^

**Figure S2:**
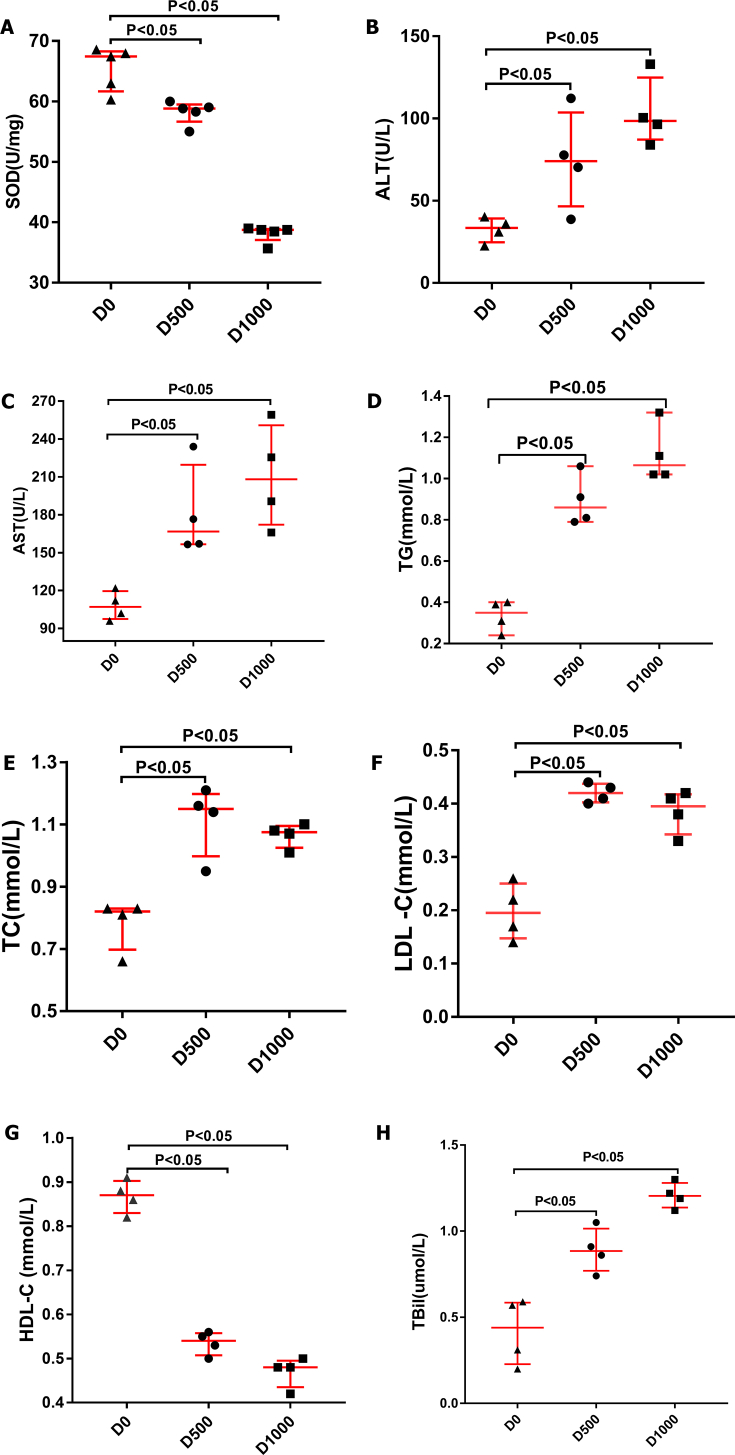
Biochemical test of liver function in the D0, D500, and D1000 groups. **(A)** Hepatic SOD levels in different groups. **(B–H)** Serum levels of ALT (B), AST (C), TG (D), TC (E), LDL-C (F), HDL-C (G), and TBil (H) in different groups. The data were presented as median with interquartile range. SOD, superoxide dismutase; ALT, alanine transaminase; AST, aspartate transaminase; TG, triglyceride; TC, total cholesterol; LDL-C, low-density lipoprotein cholesterol-c; HDL-C, high-density lipoprotein cholesterol-c; TBil, total bilirubin.

In this study, P16, P21, and P53, which are senescence markers, were highly increased in the D500 and D1000 groups (Fig. 1B–D). Additionally, the senescence-associated secretory phenotype markers Hmgb1, Mmp3, and Lamin B1 were increased in the D500 and D1000 groups (Fig. 1E). The MDA levels were increased significantly in the livers from the D500 and D1000 groups, as illustrated in Figure 1F. The hepatic antioxidant marker SOD was decreased in the D500 and D1000 groups (Fig. S2A). Biochemical tests of hepatic function revealed that the D500 and D1000 groups had decreased levels of serum high-density lipoprotein cholesterol-c and increased levels of serum alanine transaminase, aspartate transaminase, triglyceride, total cholesterol, low-density lipoprotein cholesterol-c, and total bilirubin compared with the D0 group (Fig. S2B–H). Moreover, the percentages of cells positively stained for SA-β-Gal were highly increased in the D500 and D1000 groups compared with the negative control (NC) (Fig. 1G). Some studies have found that aged livers exhibit a compromised liver regeneration capability.^2^ Hence, we performed WB experiments, which revealed that the levels of cyclin A2, cyclin B1, cyclin D1, and cyclin E1 were decreased (Fig. 1H). PCNA expression was also significantly decreased, as revealed by immunohistochemistry (Fig. 1I). Overall, the abovementioned results demonstrated that the aged liver model was successfully constructed.

Subsequently, to construct a model of senescent hepatocytes, H_2_O_2_ and D-gal were used to induce the aging of HepG2 cells. In the H_2_O_2_-induced aging model, the P16 and P21 levels were increased by H_2_O_2_ treatment (Fig. 1J, K). Considering the percentage of cell survival, treatments with 500 and 1000 μmol/L for 48 h were identified as appropriate (Fig. 1L, M). H_2_O_2_ treatment significantly increased the levels of SA-β-Gal staining in the H500 and H1000 groups (Fig. 1N). In the D-gal-induced hepatocyte aging model, the levels of P16, P21, and P53 were highly increased (Fig. 1O–Q). Based on the Cell Counting Kit-8 assay results, we selected 40 and 80 mg/mL as the doses for induction (Fig. 1R, S). Positive staining for SA-β-Gal was also found in the D40 and D80 groups (Fig. 1T). Together, these data indicated that the two hepatocyte senescence models were successfully constructed.

### RNA-seq of aged livers from rats

**Figure 2:**
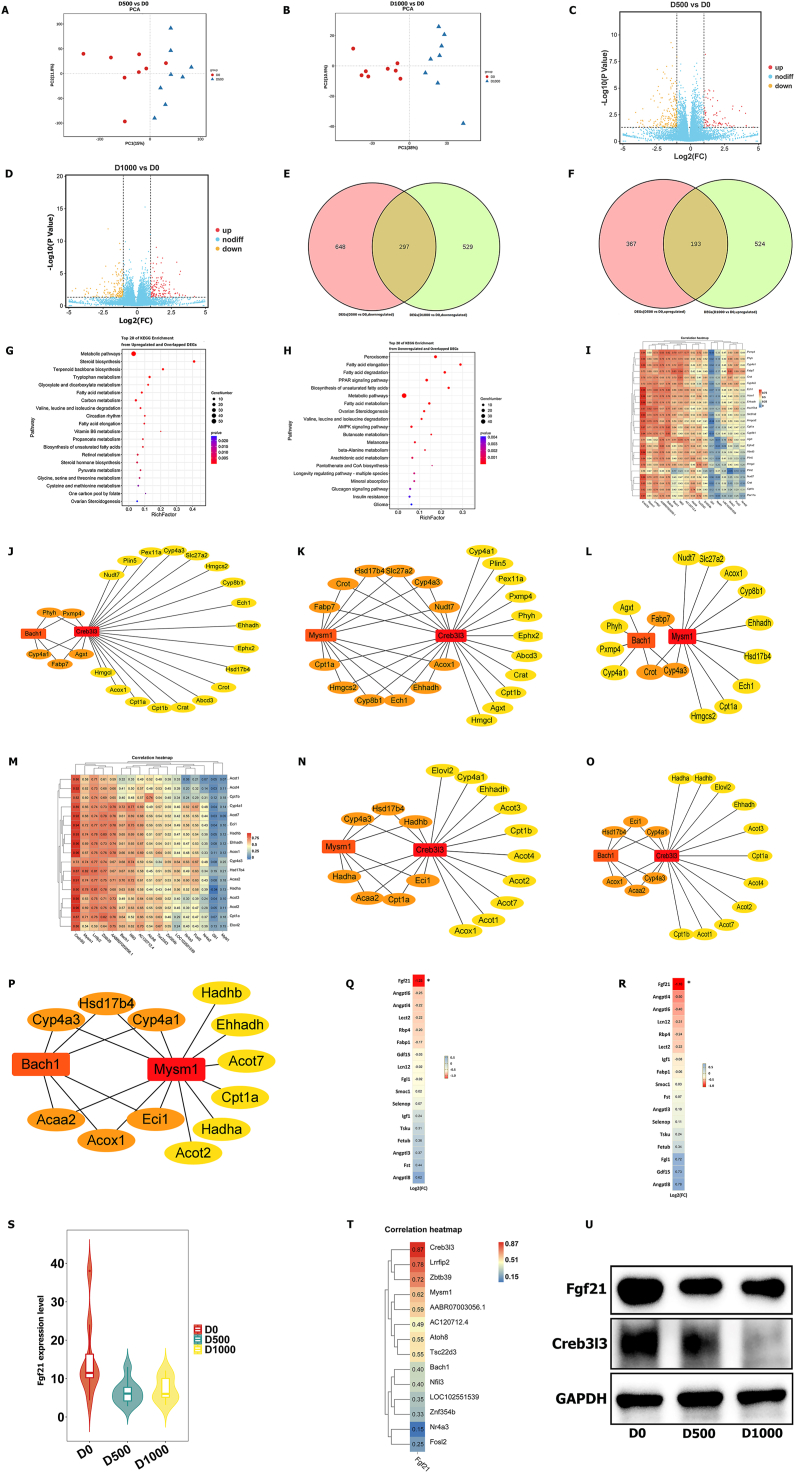
Comprehensive analysis of transcriptomic data in livers from the rat model. **(A)** PCA revealed clear separation in transcriptomic data from the livers of the D500 and D0 groups. **(B)** PCA revealed clear separation in transcriptomic data from the livers of the D1000 and D0 groups. **(C)** The volcano plot visualizing both the fold change (FC) and significance for each gene compared between the D500 and D0 groups. **(D)** The volcano plot visualizing both FC and significance for each gene compared between the D1000 and D0 groups. **(E)** The Venn diagram showing the overlapping and down-regulated DEGs between DEGs in the D500 *vs*. D0 and D1000 *vs*. D0 comparisons. **(F)** The Venn diagram showing the overlapping and up-regulated DEGs between DEGs in the D500 *vs*. D0 and D1000 *vs*. D0 comparisons. **(G)** KEGG pathway enrichment analysis for the up-regulated and overlapping DEGs. **(H)** KEGG pathway enrichment analysis for the down-regulated and overlapping DEGs. **(I)** Correlation heatmap between transcription factors (TFs) and the down-regulated DEGs enriched in peroxisomes. **(J)** Network analysis between the TFs Creb3l3, Bach1, and the key DEGs enriched in peroxisomes. Lines represent relevance. **(K)** Network analysis between the TFs Creb3l3, Mysm1, and the key DEGs enriched in peroxisomes. Lines represent relevance. **(L)** Network analysis between the TFs Bach1, Mysm1, and the key DEGs enriched in peroxisomes. Lines represent relevance. **(M)** Correlation heatmap between TFs and the down-regulated DEGs enriched in fatty acid metabolism. **(N)** Network analysis between the TFs Creb3l3, Mysm1, and the key DEGs enriched in fatty acid metabolism. Lines represent relevance. **(O)** Network analysis between the TFs Creb3l3, Bach1, and key DEGs enriched in fatty acid metabolism. Lines represent relevance. **(P)** Network analysis between the TFs Mysm1, Bach1, and the key DEGs enriched in fatty acid metabolism. Lines represent relevance. **(Q)** Log2(FC) values of hepatokines between the D500 and D0 groups. ^∗^*P* < 0.05. FC, fold change. **(R)** Log2(FC) values of hepatokines between the D1000 and D0 groups. ^∗^*P* < 0.05. FC, fold change. **(S)** Expression levels of Fgf21 in livers from the D0, D500, and D1000 groups. **(T)** Correlation heatmap between the hepatokine Fgf21 and TFs. **(U)** The expression levels of Fgf21 and Creb3l3 in livers from the D0, D500, and D1000 groups shown by western blotting.

**Figure S3:**
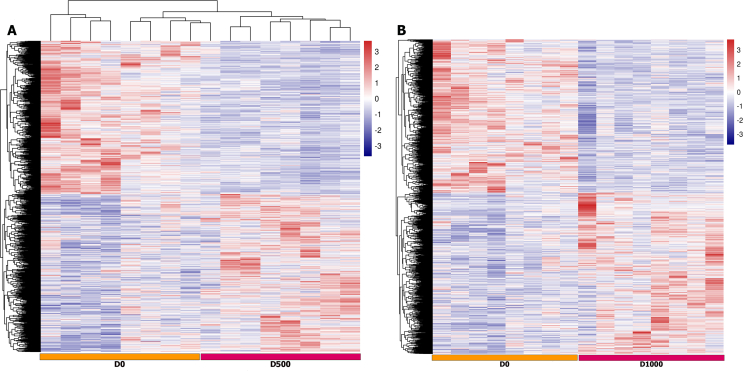
Heatmaps of DEGs in D500 vs. D0 and D1000 vs. D0. **(A)** Heatmap of DEGs between the D500 and D0 groups. **(B)** Heatmap of DEGs between the D1000 and D0 groups. DEGs, differentially expressed genes.

We sacrificed the livers of rats in the D0, D500, and D1000 groups for RNA-seq. PCA demonstrated that the transcriptomic data could be clearly separated (Fig. 2A, B), as shown in the heatmaps provided in Figure S3A and S3B. Volcano plots are shown in Figure 2C and 2D. The comparison of the D500 and D0 groups identified 945 down-regulated DEGs, and the comparison of the D1000 and D0 groups identified 826 down-regulated DEGs (Fig. 2E). Moreover, 297 down-regulated DEGs were obtained in the two abovementioned comparisons (D500 *vs*. D0 and D1000 *vs*. D0) (Fig. 2E). In addition, 560 and 717 up-regulated DEGs were obtained from the comparison of the D500 and D0 groups and the comparison of the D1000 and D0 groups, respectively (Fig. 2F). Similarly, 193 up-regulated DEGs were identified from the two abovementioned comparisons (Fig. 2F).

### Pathways of peroxisome and fatty acid metabolism are down-regulated in the aged liver and Fgf21 is pivotal in liver aging

An enrichment analysis of the DEGs was performed to explore the functional pathways of the DEGs in aged and young livers. A KEGG pathway analysis showed that the up-regulated genes in aged rats were most significantly enriched in steroid biosynthesis and terpenoid backbone biosynthesis (Fig. 2G), whereas the down-regulated genes were most significantly enriched in peroxisome and fatty acid metabolism (fatty acid elongation and degradation) (Fig. 2H). Peroxisomes are involved in lipid oxidation, and maintaining the homeostasis of peroxisomes and fatty acid metabolism is needed for longevity.^39^^,^^40^ Hence, we further analyzed the peroxisome and fatty acid metabolism pathways. The analysis found 17 TFs among the 297 down-regulated DEGs, and we performed a Pearson correlation analysis between TFs and the down-regulated genes in the peroxisome pathway (Fig. 2I). Creb3l3, Mysm1, and Bach1, which are aging-related TFs,^41^, ^42^, ^43^ were strongly correlated with the peroxisome pathway (Fig. 2I). We then constructed co-expression networks among Creb3l3, Mysm1, Bach1, and down-regulated DEGs enriched in the peroxisome pathway using Cytoscape (*P* < 0.05), and these results indicated that Creb3l3 was more strongly correlated with peroxisome genes than Mysm1 and Bach1 (Fig. 2J–L). We further analyzed the enrichment pathway of fatty acid metabolism and similarly performed Pearson correlation analysis between TFs and the down-regulated genes of fatty acid metabolism (Fig. 2M). Similarly, we constructed co-expression networks of Creb3l3, Mysm1, Bach1, and the down-regulated DEGs enriched in fatty acid metabolism with Cytoscape (*P* < 0.05), and the results indicated that Creb3l3 was still the most important TF in fatty acid metabolism (Fig. 2N–P). Creb3l3 regulates lipophagy, the oxidation of fatty acids, and the expression of apolipoproteins to maintain the homeostasis of lipid metabolism by governing related gene expression.^43^ Notably, fatty acid accumulation can trigger aging of the liver.^12^ Hence, Creb3l3 is an important TF worth further research. Overall, these results demonstrate that Creb3l3 plays an important role in regulating peroxisome and fatty acid metabolism during liver aging.

Transcriptomic data identified a total of 17 hepatocyte growth factors. Only the expression of Fgf21 differed among the D0, D500, and D1000 groups (*P* < 0.05; Fig. 2Q, R); Fgf21 expression was significantly lower in the aged livers of the D500 and D1000 groups than in the livers of the D0 group (*P* < 0.05; Fig. 2S). We then conducted a Pearson correlation analysis between Fgf21 and TFs (Fig. 2T). The results indicated that Creb3l3 was significantly and positively co-expressed with Fgf21 (Fig. 2T). Some previous studies have found that Fgf21 plays important roles in regulating lipid metabolism and extending the lifespan.^11^^,^^12^ Our WB results further demonstrated that Fgf21 and Creb3l3 expression were lower in the livers of the D500 and D1000 groups than in those of the D0 group (Fig. 2U). Overall, these results demonstrated that Fgf21 played an important role in Creb3l3-mediated regulation of peroxisomal function and fatty acid metabolism during liver aging.

### Metabolomic analysis of aged livers

**Figure 3:**
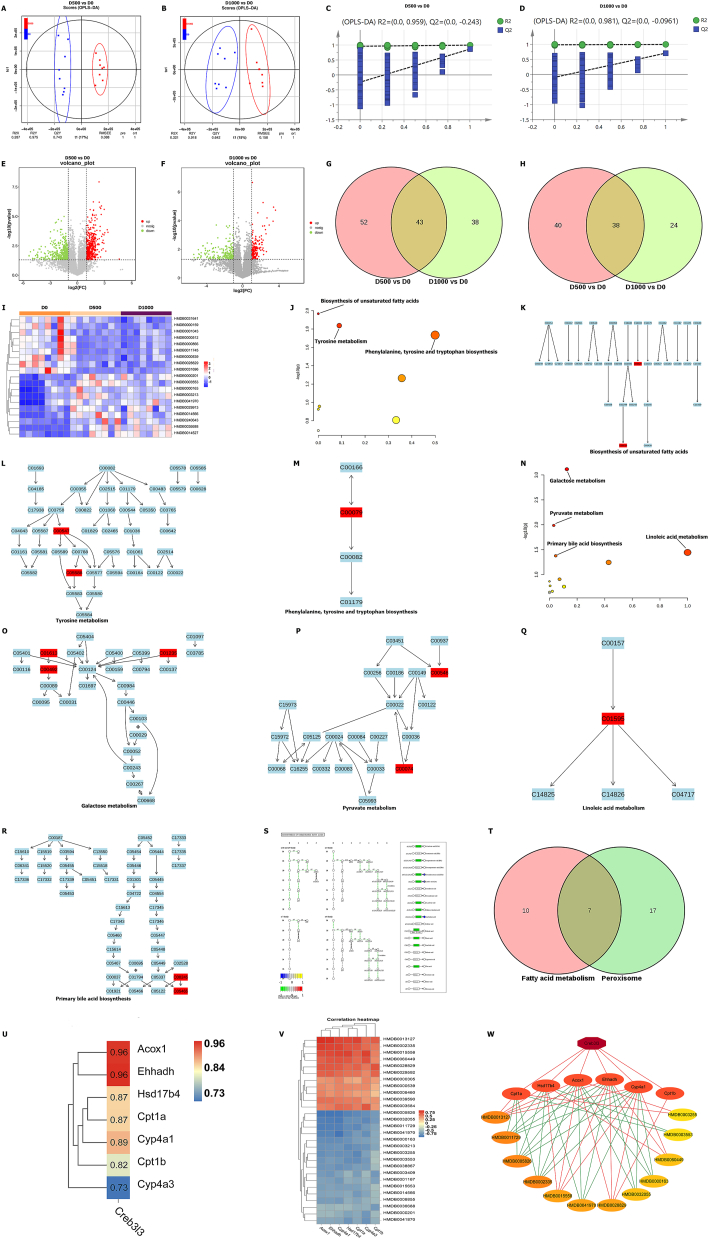
Comprehensive analysis of metabolomic data from livers in the rat model. **(A)** PCA revealed clear separation in metabolomic data from the livers of the D500 and D0 groups. **(B)** PCA revealed clear separation in metabolomic data from the livers of the D1000 and D0 groups. **(C)** Validation of the OPLS-DA model by class permutation analysis for (A). **(D)** Validation of the OPLS-DA model by class permutation analysis for (B). **(E)** The volcano plot visualizing both the FC and significance for each metabolite compared between the D500 and D0 groups. **(F)** The volcano plot visualizing both the FC and significance for each metabolite compared between the D1000 and D0 groups. **(G)** The Venn diagram showing the overlapping and down-regulated DEMs between DEMs in the D500 *vs*. D0 and D1000 *vs*. D0 comparisons. **(H)** The Venn diagram showing the overlapping and up-regulated DEMs between DEMs in the D500 *vs*. D0 and D1000 *vs*. D0 comparisons. **(I)** Heatmap for the overlapping DEMs in the D0, D500, and D1000 groups. **(J)** Enrichment pathway analysis for overlapping and down-regulated DEMs. **(K)** Details of the pathway for the biosynthesis of unsaturated fatty acids. Red represents down-regulated metabolites. **(L)** Details of the pathway for tyrosine metabolism. Red represents down-regulated metabolites. **(M)** Details of the pathway for phenylalanine, tyrosine, and tryptophan biosynthesis. Red represents down-regulated metabolites. **(N)** Enrichment pathway analysis for overlapping and up-regulated DEMs. **(O)** Details of the galactose metabolism pathway. Red represents up-regulated metabolites. **(P)** Details of the pyruvate metabolism pathway. Red represents up-regulated metabolites. **(Q)** Details of the linoleic acid metabolism pathway. Red represents up-regulated metabolites. **(R)** Details of the primary bile acid biosynthesis pathway. Red represents up-regulated metabolites. **(S)** Integrative transcriptomic and nontargeted metabolomic analysis identifies variations in pathways of biosynthesis of unsaturated fatty acids in aged livers. **(T)** The Venn diagram showing the overlapping and down-regulated DEMs between the peroxisome and fatty acid metabolism pathways. **(U)** Correlation heatmap between Creb3l3 and key DEGs. DEGs, differentially expressed genes. **(V)** Correlation heatmap between these key genes and key DEMs. **(W)** Co-expression network among Creb3l3, DEGs, and DEMs. Red lines represent positive correlations (*P* < 0.05), and green lines represent negative correlations (*P* < 0.05). DEGs, differentially expressed genes; DEMs, differentially expressed metabolites.

**Figure S4:**
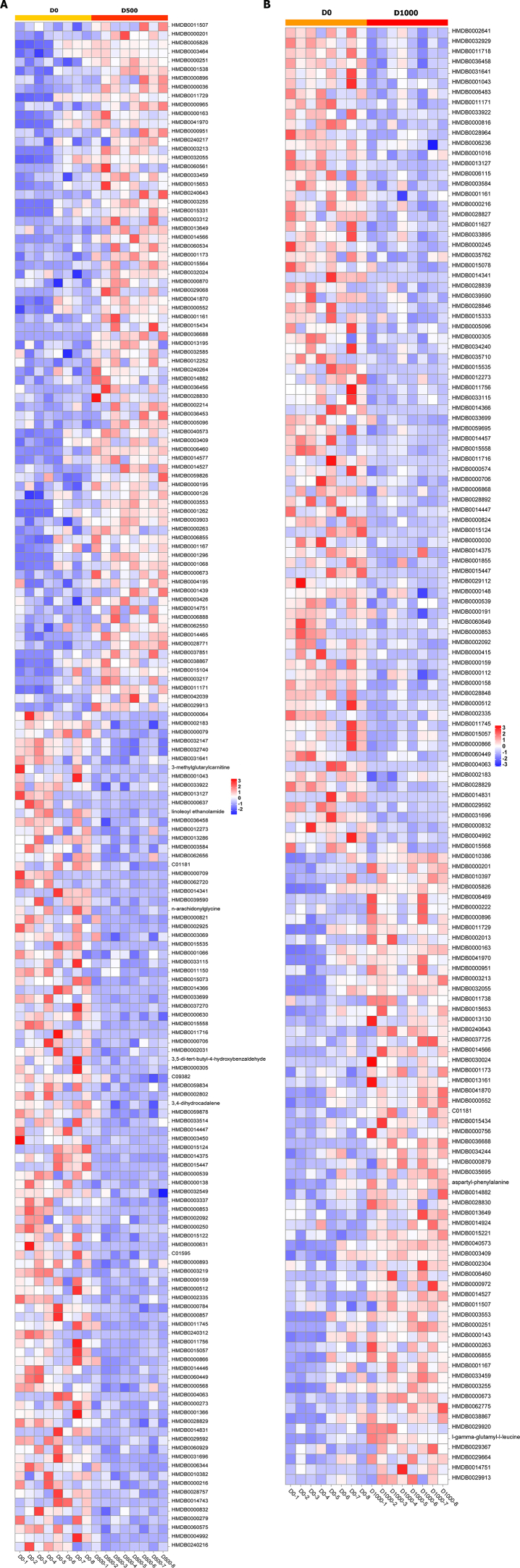
Heatmaps of DEMs in D500 vs. D0 and D1000 vs. D0. **(A)** Heatmap of DEMs between the D500 and D0 groups. **(B)** Heatmap of DEMs between the D1000 and D0 groups. DEMs, differentially expressed metabolites.

The whole–metabolomic profiles of livers belonging to the D0, D500, and D1000 groups were assayed by nontargeted metabolomic analysis. The metabolomic profiles of the D500 *vs*. D0 and D1000 *vs*. D0 comparisons could be separated by OPLS-DA (Fig. 3A, B). These results were validated by permutation analysis (*R*2 = 0.959, *Q*2 = −0.243 for D500 *vs*. D0; *R*2 = 0.981, *Q*2 = −0.0961 for D1000 *vs*. D0; Fig. 3C, D). The comparison between the D500 and D0 groups identified 95 down-regulated DEMs and 78 up-regulated DEMs (Fig. 3E; Fig. S4A), whereas 81 down-regulated DEMs and 62 up-regulated DEMs were found from the comparison between the D1000 and D0 groups (Fig. 3F; Fig. S4B). We then identified 43 down-regulated DEMs and 38 up-regulated DEMs in the intersection of the DEMs identified from the D500 *vs*. D0 and D1000 *vs*. D0 comparisons (Fig. 3G, H). These intersecting and key DEMs are presented in a heatmap (Fig. 3I).

We subsequently performed a pathway analysis with MetaboAnalyst 5.0 to further identify the key pathways and metabolites. The pathway analysis demonstrated that biosynthesis of unsaturated fatty acids, tyrosine metabolism, and phenylalanine, tyrosine, and tryptophan biosynthesis were all significantly enriched in the down-regulated and overlapping DEMs (*P* < 0.05; Fig. 3J). Regarding the biosynthesis of unsaturated fatty acids, docosahexaenoic acid and arachidonic acid were significantly decreased (*P* < 0.05; Fig. 3K). Additionally, regarding tyrosine metabolism and phenylalanine, tyrosine and tryptophan biosynthesis, phenols, carboxylic acids, and derivatives were significantly decreased (*P* < 0.05; Fig. 3L, M). The pathway analysis of the up-regulated and overlapping DEMs revealed that galactose metabolism, pyruvate metabolism, linoleic acid metabolism, and primary bile acid biosynthesis were significantly enriched (*P* < 0.05; Fig. 3N). Organooxygen compounds were enriched significantly in galactose metabolism (*P* < 0.05; Fig. 3O). Additionally, organooxygen compounds and organic phosphoric acids and derivatives were enriched in pyruvate metabolism (*P* < 0.05; Fig. 3P). Fatty acyls were enriched in linoleic acid metabolism, and organic sulfonic acids and derivatives, and steroids and steroid derivatives were enriched in primary bile acid biosynthesis (*P* < 0.05; Fig. 3Q, R).

### Down-regulation of Fgf21 is important for the transcription-metabolism regulatory network in aged livers

According to the abovementioned illustrated transcriptomic and metabolomic data, we performed a multiomics analysis by constructing a transcription-metabolism regulatory network. First, we performed a joint pathway analysis of the down-regulated DEGs and overlapping DEMs, and the results showed that biosynthesis of unsaturated fatty acids was down-regulated (*P* < 0.05; Fig. 3S). Because Creb3l3 plays a vital role in peroxisomes and fatty acid metabolism, we identified seven key genes by intersecting the peroxisome and fatty acid metabolism pathways (Fig. 3T). We conducted a Pearson correlation analysis between Creb3l3 and these key genes, and the results are shown in Figure 3U. Additionally, we conducted a correlation analysis between these key genes and the overlapping DEMs (*P* < 0.05; Fig. 3V). Overall, we obtained a co-expression network between transcriptomics and metabolomics (Fig. 3W). We previously also found that Fgf21 expression was strongly correlated with Creb3l3 expression and thus speculated that Fgf21 is also involved in regulating the transcriptomics-metabolomics co-expression network.

### Transcriptomic analysis of senescent hepatocytes

**Figure 4:**
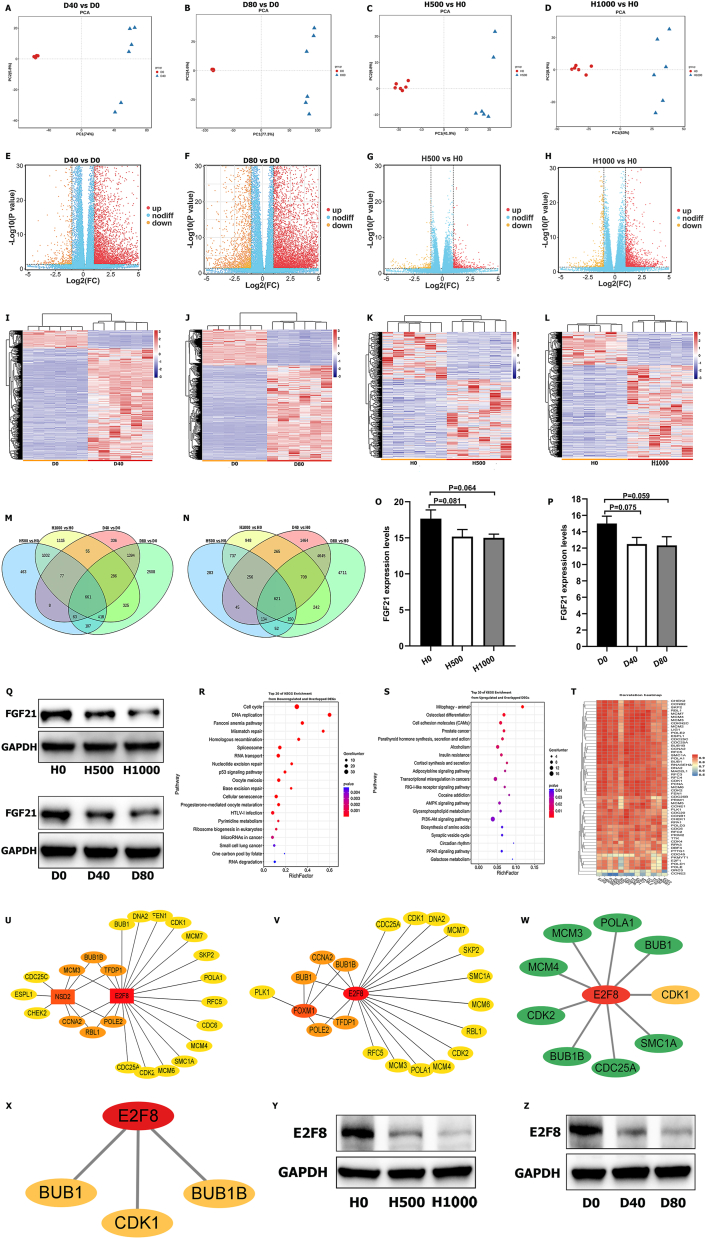
Comprehensive analysis of transcriptomic data in a model of senescent hepatocytes. **(A‒D)** PCA revealed a clear separation in transcriptomic data from the D40/D0 groups (A), the D80/D0 groups (B), the H500/H0 groups (C), and the H1000/H0 groups (D). **(E)** The volcano plot visualizing both the FC and significance for each gene compared between the D40 and D0 groups. **(F)** The volcano plot visualizing both the FC and significance for each gene compared between the D80 and D0 groups. **(G)** The volcano plot visualizing both the FC and significance for each gene compared between the H500 and H0 groups. **(H)** The volcano plot visualizing both the FC and significance for each gene compared between the H1000 and H0 groups. **(I‒L)** Heatmap for DEGs between the D40 and D0 groups (I), between the D80 and D0 groups (J), between the H500 and H0 groups (K), and between the H1000 and H0 groups (L). **(M)** The Venn diagram showing the overlapping and down-regulated DEGs in the senescent hepatocytes. **(N)** The Venn diagram showing the overlapping and up-regulated DEGs in the senescent hepatocytes. **(O)** Expression levels of Fgf21 in livers from the H0, H500, and H1000 groups. **(P)** Expression levels of Fgf21 in livers from the D0, D40, and D80 groups. **(Q)** WB for the expression levels of FGF21 in hepatocytes treated with H_2_O_2_ and D-gal, respectively. **(R)** Enrichment pathway analysis for overlapping and down-regulated DEGs. **(S)** Enrichment pathway analysis for overlapping and up-regulated DEGs. **(T)** Correlation heatmap between TFs and DEGs enriched in the cell cycle. TF, transcription factor. **(U)** Network analysis between the E2F8, NSD2, and overlapping DEGs enriched in the cell cycle. Lines represent relevance. **(V)** Network analysis between the E2F8, FOXM1, and overlapping DEGs enriched in the cell cycle. Lines represent relevance. **(W)** Network analysis between E2F8 and overlapping DEGs enriched in the cell cycle. Green lines represent E2F8 regulating the expression of genes by TFBSs. **(X)** Protein–protein interaction network between E2F8 and genes. **(Y)** WB for the expression levels of E2F8 in hepatocytes treated with H_2_O_2_. **(Z)** WB for the expression levels of E2F8 in hepatocytes treated with D-gal. WB, western blotting; TFBSs, transcription factor-binding sites; DEGs, differentially expressed genes.

The whole–transcriptome profiles of the D0, D40, D80, H0, H500, and H1000 groups were then assayed by RNA-seq. The transcriptomic data were separated by PCA into these groups (Fig. 4A–D), and the volcano plots and heatmap are shown in Figure 4E–L. Overall, 2829, 3969, 2880, and 5762 down-regulated DEGs were identified from the H500 *vs*. H0, H1000 *vs*. H0, D40 *vs*. D0, and D80 *vs*. D0 comparisons, respectively (Fig. 4M), and we obtained 661 down-regulated DEGs in the intersection of these groups (Fig. 4M). Additionally, 2278, 3928, 8139, and 11264 up-regulated DEGs were obtained from the H500 *vs*. H0, H1000 *vs*. H0, D40 *vs*. D0, and D80 *vs*. D0 comparisons, respectively (Fig. 4N), and their intersection comprised 621 up-regulated DEGs (Fig. 4N).

### The expression of FGF21 is down-regulated in senescent hepatocytes, and senescent hepatocytes exhibit a compromised regenerative ability

FGF21 plays a vital role in the liver aging of rats, and we thus studied the role of FGF21 in senescent hepatocytes. Although the difference in FGF21 expression between the senescent and control hepatocytes was not significant (H500 *vs*. H0: *P* = 0.081, H1000 *vs*. H0: *P* = 0.064, D40 *vs*. D0: *P* = 0.075, D80 *vs*. D0: *P* = 0.059), a decreasing trend in the expression of FGF21 was found in senescent hepatocytes compared with the control cells (Fig. 4O, P). We then performed a WB analysis of senescent hepatocytes, and similar to the results found for aged rat livers, the expression of FGF21 was decreased in senescent compared with control hepatocytes (Fig. 4Q). These results demonstrated that FGF21 plays an important role in liver aging.

We subsequently performed enrichment analyses of these 661 down-regulated DEGs and 621 up-regulated DEGs, and the results are shown in Figure 4R, S. These down-regulated DEGs were enriched significantly in the cell cycle and DNA replication, and the up-regulated DEGs were enriched in mitophagy. The significance and number of the DEGs enriched in mitophagy were far lower than those enriched in the cell cycle and DNA replication, and some studies have reported that the cell cycle is inhibited in aged hepatocytes.^44^^,^^45^ Thus, we further researched the cell cycle and DNA replication, and indeed, DNA replication was also part of the cell cycle. Additionally, the down-regulated DEGs included 13 TFs, and we thus performed a Pearson correlation analysis between these TFs and the down-regulated DEGs involved in the cell cycle (Fig. 4T). Previous studies have found that E2F8, FOXM1, and NSD2 are aging-related proteins.^46^^,^^47^ In this study, we found that E2F8, FOXM1, and NSD2 were positively correlated with the down-regulated DEGs and that E2F8 was also involved in the cell cycle pathway and played an essential regulatory role in this process. We constructed a co-expression network among E2F8, FOXM1, NSD2, and the down-regulated genes involved in the cell cycle using Cytoscape (*P* < 0.05; Fig. 4U, V). These results showed that E2F8 was the most positively correlated with the cell cycle. Hence, we speculated that E2F8 could regulate the expression of genes that play key roles in the cell cycle, and we thus constructed a co-expression network between E2F8 and key genes using Cytoscape (RR > 0.97; Fig. 4W). A Jaspar analysis found that E2F8 closely regulated the expression of most of these key genes (Table 1). In addition, protein–protein interaction networks further showed that BUB1, BUB1B, and CDK1 were closely related to E2F8 (Fig. 4X). A WB analysis also demonstrated that E2F8 was significantly decreased in aged hepatocytes (Fig. 4Y, Z).

**Table 1:** Information about the genes whose expression is regulated by the transcription factor E2F8.

Genes	TF	Score	Start	End	Strand	Predicted sequence
MCM4	E2F8	16.01	1772	1783	+	TTCCCGCGAAAA
MCM3	E2F8	15.10	1973	1984	–	TTCCCGCCACCA
POLA1	E2F8	9.60	147	158	–	CGCCCGCCACCA
BUB1	E2F8	9.94	611	622	–	TTGGCGCCACCA
SMC1A	E2F8	11.58	1545	1556	+	GTCCCGCCACCA
CDC25A	E2F8	10.49	1956	1967	–	TTGGCGCCAAAC
BUB1B	E2F8	9.69	1984	1995	–	TAGCCGCCAAGT
CDK2	E2F8	8.81	1938	1949	–	TTCCCGCGTTTC

TF, transcription factor.

### FGF21 attenuates liver aging

**Figure 5:**
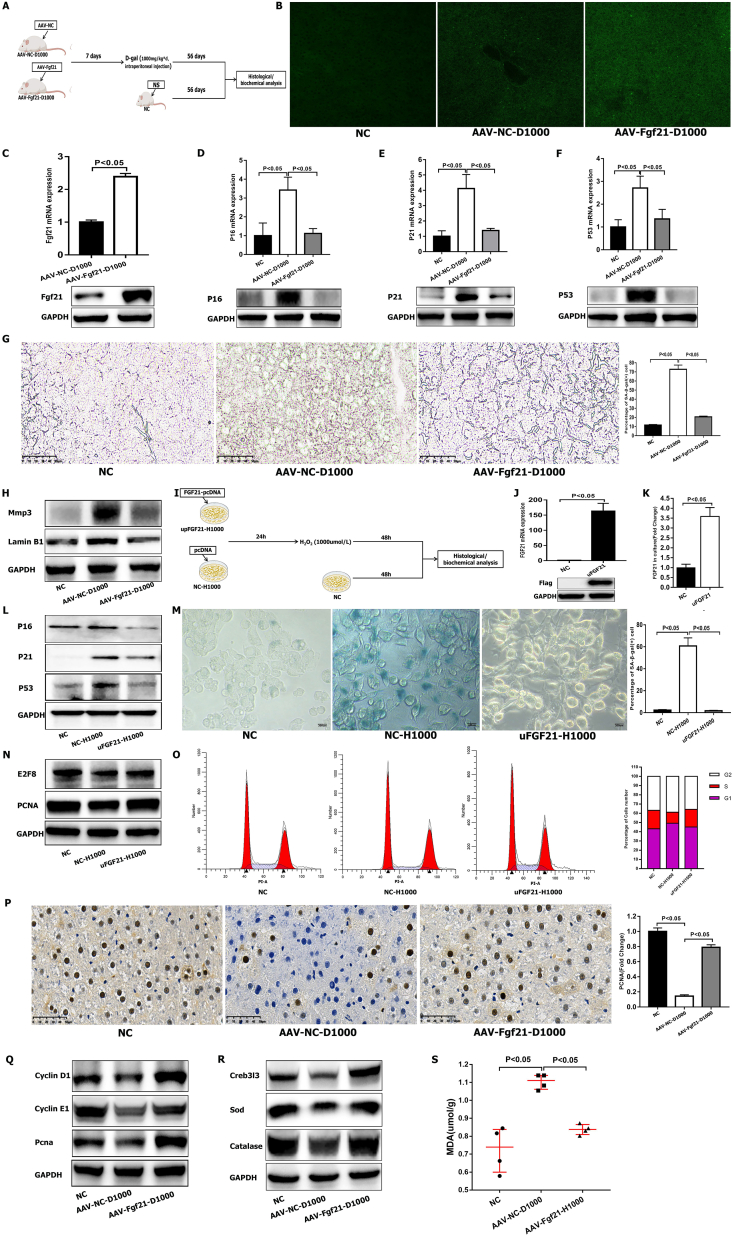
Up-regulation of the expression of FGF21 in the induced aged liver and senescent hepatocyte models. **(A)** Rats treated with AAV and subjected to induced liver aging by D-gal. AAV, adeno-associated virus. **(B)** Expression of GFP in livers from rats. GFP, green fluorescent protein. **(C–F)** RT‒qPCR for the expression levels of Fgf21 (C), P16 (D), P21 (E), and P53 (F) in livers. The data were presented as mean ± standard error of the mean (SEM). Their protein levels were validated by Western blotting (WB). **(G)** SA-β-Gal staining in livers. The data were presented as mean ± SEM. **(H)** WB for the expression levels of Mmp3 and Lamin B1 in livers. **(I)** Up-regulating the expression of FGF21 by plasmid and inducing the model of senescent hepatocytes. **(J)** RT‒qPCR for the expression levels of FGF21 in cells. The data were presented as mean ± SEM. The FGF21 protein levels were validated by WB. **(K)** ELISA experiment for the levels of FGF21 in the culture medium of cells. **(L)** WB for the expression levels of P16, P21, and P53. **(M)** SA-β-Gal staining in cells treated with H_2_O_2_. The data were presented as mean ± SEM. **(N)** WB for the expression levels of E2F8 and PCNA in cells from the NC, NC-H1000, and uFGF21-H1000 groups. **(O)** Flow cytometry analysis of the cell cycle in the NC, NC-H1000, and uFGF21-H1000 groups. **(P)** Immunohistochemical staining for the expression of Pcna in livers from the NC, AAV–NC–D1000, and AAV-Fgf21-D1000 groups. The data were presented as mean ± SEM. **(Q)** WB for the levels of Cyclin D1, Cyclin E1, and Pcna in livers from the NC, AAV–NC–D1000, and AAV-Fgf21-D1000 groups. The data were presented as mean ± SEM. **(R)** WB for the levels of Creb3l3, Sod, and Catalase in livers from the NC, AAV–NC–D1000, and AAV-Fgf21-D1000 groups. The data were presented as mean ± SEM. **(S)** The levels of MDA in livers from the NC, AAV–NC–D1000, and AAV-Fgf21-D1000 groups. The data were presented as median with interquartile range. MDA, malondialdehyde.

**Figure S5:**
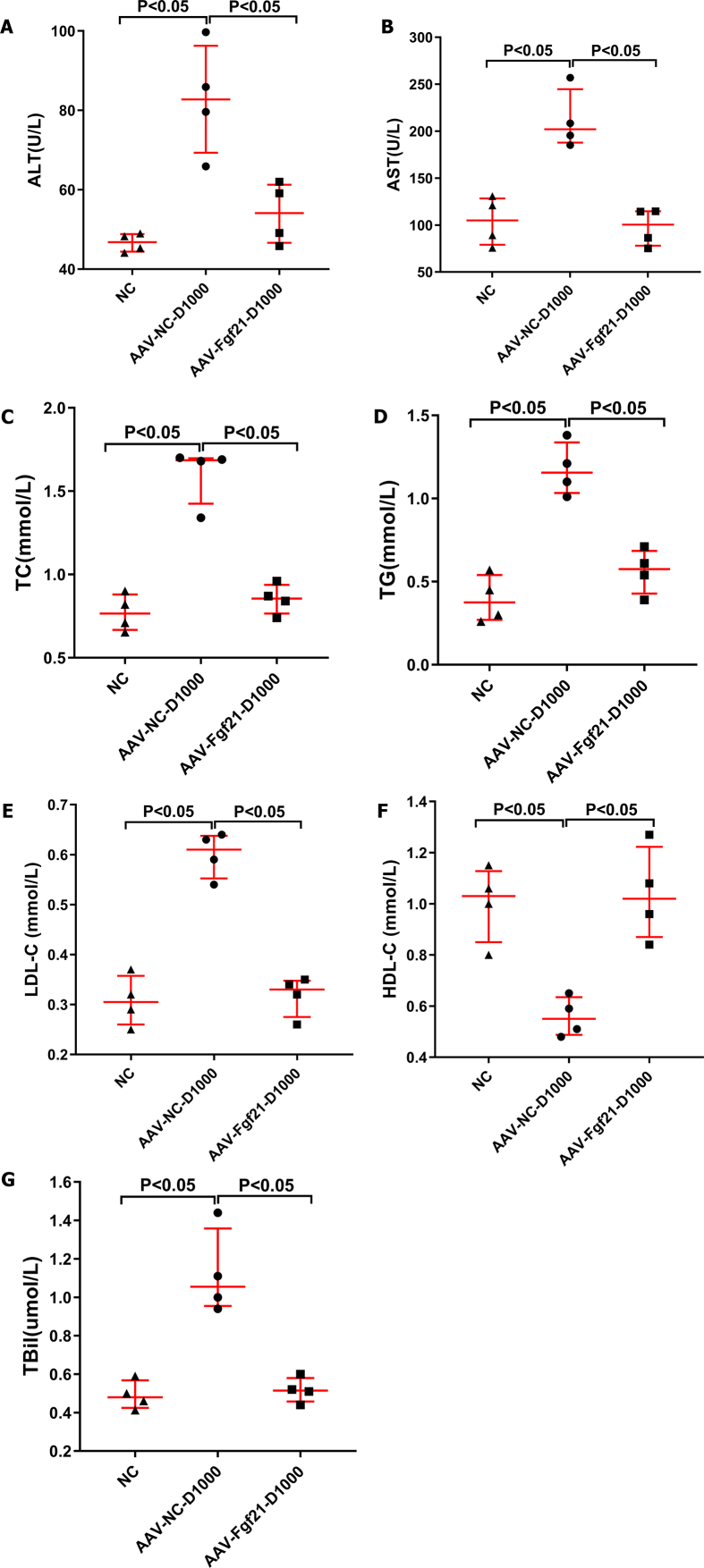
Biochemical test of liver function in the NC, AAV–NC–D1000, and AAV-Fgf21-D1000 groups. **(A‒G)** Serum levels of ALT (A), AST (B), TC (C), TG (D), LDL-C (E), HDL-C (F), and TBil (G) in different groups. The data were presented as median with interquartile range. ALT, alanine transaminase; AST, aspartate transaminase; TC, total cholesterol; TG, triglyceride; LDL-C, low-density lipoprotein cholesterol; HDL-C, high-density lipoprotein cholesterol; TBil, total bilirubin.

Because the RNA-seq findings demonstrated that FGF21 plays a vital role in liver aging, we overexpressed FGF21 in the cells and livers of rats using plasmids and adeno-associated viruses (AAV), respectively. First, 24 rats were divided into the NC, AAV–NC–D1000, and AAV-Fgf21-D1000 groups (Fig. 5A). The rats in the AAV–NC–D1000 and AAV-Fgf21-D1000 groups were then infected with AAV-NC and AAV-Fgf21, respectively. After 1 week, the rats in these two groups were injected with D-gal (1000 mg/kg per day) for 8 weeks (Fig. 5A). The rats in the NC group were injected with normal saline solution for 8 weeks (Fig. 5A). We sacrificed the rats and obtained their livers to perform histological and biochemical analyses. The AAVs contained the green fluorescent protein gene which was more highly expressed in the AAV–NC–D1000 and AAV-Fgf21-D1000 groups compared with the NC group (Fig. 5B). It showed that the AAVs successfully infected the liver. In addition, the WB and PCR analyses showed that Fgf21 was highly expressed in the AAV-Fgf21-D1000 group (Fig. 5C). We then examined senescence markers and found that the P16, P21, and P53 levels were significantly lower in the livers of rats in the AAV-Fgf21-D1000 group than in those of rats in the AAV–NC–D1000 group (Fig. 5D–F). In the AAV-Fgf21-D1000 group, the percentage of SA-β-Gal-stained (+) cells was decreased (Fig. 5G), and the levels of the senescence markers Mmp3 and Lamin B1 were significantly reduced (Fig. 5H). These results indicated that Fgf21 can inhibit liver aging. In addition, the AAV-Fgf21-D1000 group showed decreased levels of serum alanine transaminase, aspartate transaminase, total cholesterol, triglyceride, low-density lipoprotein cholesterol-c, and total bilirubin and higher levels of high-density lipoprotein cholesterol-c, which showed that the function of the liver was improved (Fig. S5A–G). We then up-regulated the expression of FGF21 in HepG2 cells using the FGF21-pcDNA plasmid to study the effect of this protein against liver aging (Fig. 5I). HepG2 cells in the upFGF21-H1000 and NC-H1000 groups were transfected with FGF21-pcDNA and pc-DNA, respectively, and then treated with H_2_O_2_ (1000 μmol/L) for 48 h to induce aging (Fig. 5I). The cells in the NC group, which were only cultured for 48 h, were regarded as controls (Fig. 5I). PCR and WB analyses indicated that FGF21 was highly expressed in the cells transfected with FGF21-pcDNA (Fig. 5J). In addition, the FGF21 levels in the culture supernatant of the HepG2 cells were higher than those in the control cell supernatant, as revealed by ELISA (Fig. 5K). We then found that the expression of the senescence markers P16, P21, and P53 was significantly lower in HepG2 cells transfected with FGF21-pcDNA than in NC-H1000 cells (Fig. 5L). Moreover, the percentage of SA-β-Gal-stained (+) HepG2 cells transfected with FGF21-pcDNA was lower than that of NC-H1000 cells (Fig. 5M). Overall, these results obtained from experiments in rats and cells demonstrate that FGF21 can protect against liver aging.

### FGF21 inhibits the declines in the regenerative and antioxidant capacities that occur during liver aging

The expression of E2F8 was increased significantly in the upFGF21-H1000 group of HepG2 cells (Fig. 5N). PCNA expression was also higher in the upFGF21-H1000 group than in the NC-H1000 group (Fig. 5N). Flow cytometry analysis of the cell cycle revealed that fewer cells in the NC-H1000 group entered the S phase, but the number of cells in the S phase was increased in the upFGF21-H1000 group (Fig. 5O). The expression of P16, P21, and P53, which are cell cycle inhibitors, was also decreased in the upFGF21-H1000 group (Fig. 5L). Immunohistochemical staining demonstrated that Pcna was more highly expressed in the livers of the AAV-FGF21-D1000 group compared with the AAV–NC–D1000 group (Fig. 5P). In addition, in the rat model (Fig. 5A), cyclin D1, cyclin E1, and PCNA in the livers of rats treated with AAV-FGF21 were significantly greater than those in the livers of rats treated with AAV–NC–D1000 (Fig. 5Q). Overall, these results indicated that FGF21 could inhibit the decline in regenerative ability that occurs during liver aging.

We subsequently found that Creb3l3 was highly expressed in the AAV-FGF21-D1000 group (Fig. 5R), which indicated that it was co-expressed with Fgf21. Additionally, the levels of SOD and catalase were higher in the AAV-Fgf21-D1000 group than in the AAV–NC–D1000 group (Fig. 5R). We found that the levels of the lipid peroxidation product MDA were increased in the AAV–NC–D1000 group but decreased in the AAV-Fgf21-D1000 group than that found in the NC group (Fig. 5S). Hence, the livers of rats treated with AAV-Fgf21 exhibited an increased antioxidant capability compared with those of the AAV–NC–D1000 group. Overall, Fgf21 can also inhibit the decline in antioxidant ability that occurs during the process of liver aging.

### Validation of the results from animal and cellular experiments using human liver and blood samples

**Figure 6:**
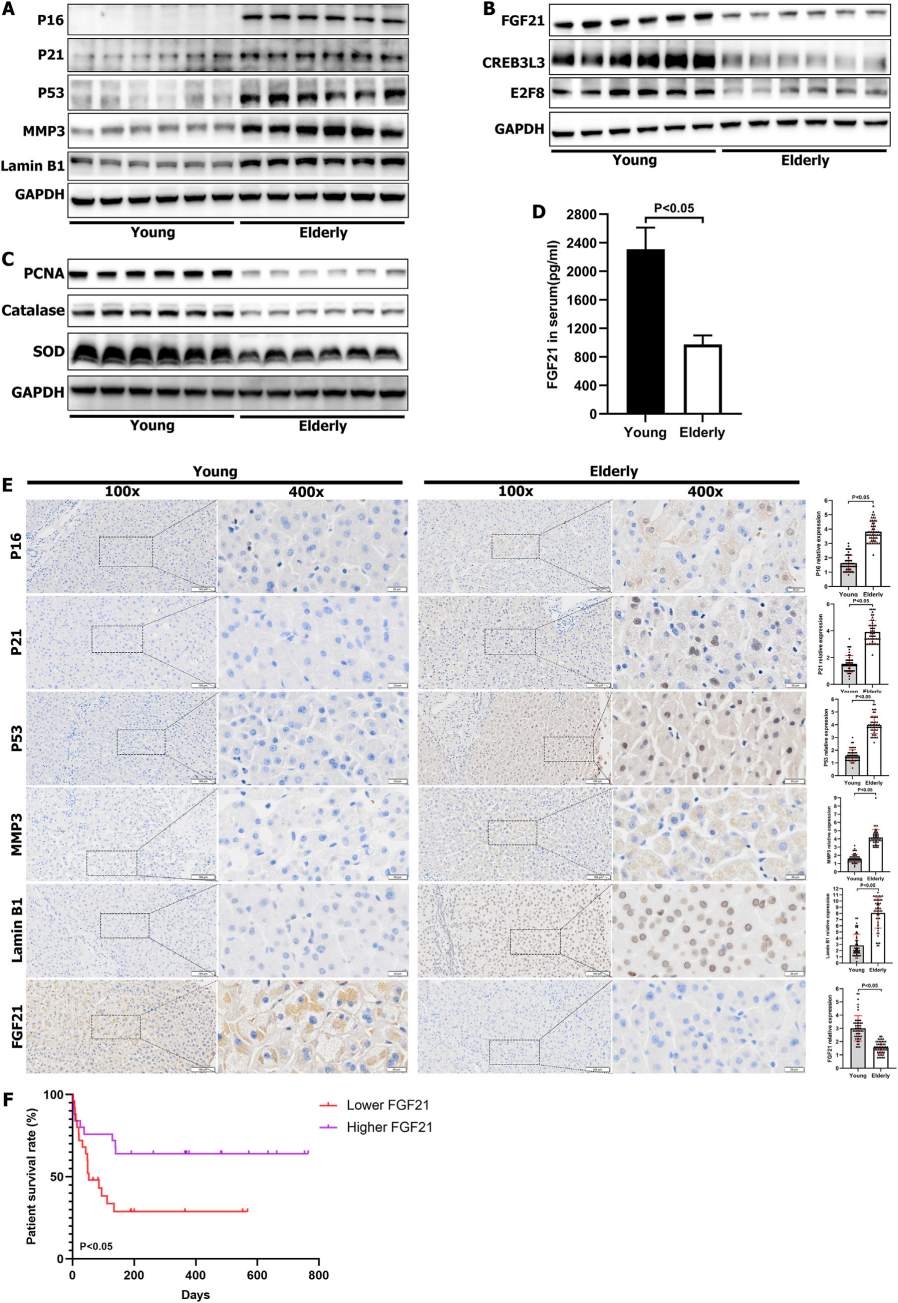
Validation of the results of cellular and animal experiments in human livers. **(A)** The expression levels of P16, P21, P53, MMP3, and Lamin B in the livers from humans shown by western blotting (WB). **(B)** The expression levels of FGF21, CREB3L3, and E2F8 in the livers from humans shown by WB. **(C)** The expression levels of PCNA, catalase, and SOD in the livers from humans shown by WB. **(D)** ELISA experiment demonstrated that FGF21 of serum was decreased significantly in the elderly group. **(E)** Immunohistochemical staining of P16, P21, P53, MMP3, Lamin B1, and FGF21 in human liver tissue microarrays. **(F)** The survival rate of patients performed with liver transplantation using older grafts categorized by FGF21 expression levels (lower FGF21: immunohistochemical staining scores ≤1.6; higher FGF21: immunohistochemical staining scores ≥1.8). Young represents the livers of people less than 50 years old; Elderly represents the livers of people over 60 years old.

**Figure S6:**
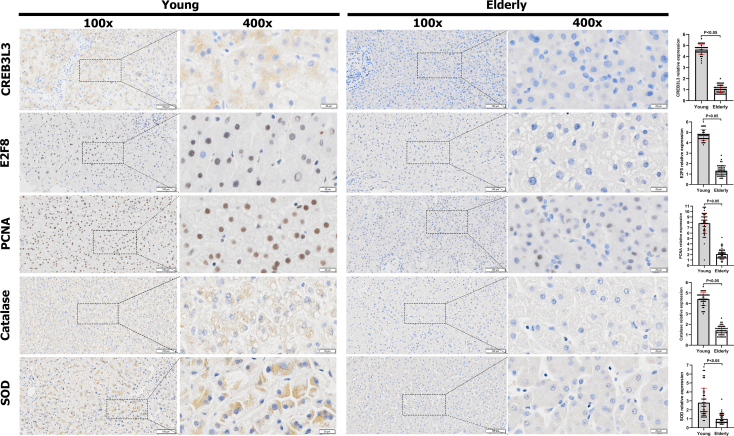
Immunohistochemical staining of CREB3L3, E2F8, PCNA, catalase, and SOD in human liver tissue microarrays. SOD, superoxide dismutase.

To verify our findings using human samples, we obtained liver specimens and peripheral blood from younger and elderly humans. Samples from people older than 60 years of age comprised the elderly group. We examined the expression levels of P16, P21, P53, MMP3, and Lamin B1, which are all markers of senescence. Similar to our findings in rats and cells, these markers were more highly expressed in the aged livers of humans (Fig. 6A). Subsequently, FGF21 was significantly decreased in the livers of elderly individuals (Fig. 6B). Moreover, the levels of CREB3L3 and E2F8 were also decreased in aged livers (Fig. 6B), and the levels of PCNA, catalase, and SOD were decreased in the livers of older individuals (Fig. 6C), indicating that the regenerative and antioxidant capabilities were decreased in aged livers. Moreover, the serum FGF21 level was also lower in the elderly, as revealed by ELISA (Fig. 6D). Next, we conducted human liver immunohistochemical staining to validate the results obtained from rats and cells. Results revealed that the levels of the senescence markers P16, P21, P53, MMP3, and Lamin B1 were increased in the livers of the elderly group (Fig. 6E). Similarly, the FGF21, CREB3L3, and E2F8 levels were decreased in the livers of aged humans (Fig. 6E; Fig. S6). In addition, the PCNA, catalase, and SOD levels were decreased (Fig. 6E; Fig. S6), which indicated declines in the regenerative and antioxidant abilities of the livers of elderly individuals. According to the relative expressions of donor liver FGF21 immunohistochemical staining in the elderly donor group, we classified the recipients into the lower donor liver FGF21 expression group (Lower FGF21: immunohistochemical staining scores ≤1.6) and the higher donor liver FGF21 expression group (Higher FGF21: immunohistochemical staining scores ≥1.8) to determine the importance of FGF21 expression in the prognosis of liver transplantation. The results showed that the recipients with lower donor liver FGF21 expression exhibited lower survival than the control group (Fig. 6F). Overall, these results further validated our findings from animal and cellular experiments, which indicated that FGF21 is an important target to inhibit the liver aging process.

## Discussion

Liver aging is a contributor to chronic liver diseases, including nonalcoholic fatty liver disease and liver fibrosis, and livers from older donors have a high rate of graft failure after liver transplantation.^2^^,^^48^ Hence, it is essential to clarify the underlying key mechanism of liver aging and identify therapeutic targets to attenuate the damage caused by liver aging. To address these needs, we first constructed models of aged livers with D-gal. Two other types of models of senescent hepatocytes were established with H_2_O_2_ and D-gal. We subsequently performed transcriptomic and metabolomic analyses of these models. The RNA-seq results from the aged liver model indicated that genes associated with peroxisomes and fatty acid metabolism were significantly down-regulated and that the decreases in the expression of Fgf21 and Creb3l3 were positively related to the decreases in the expression of these genes. Moreover, a multi-omics analysis showed that genes related to the biosynthesis of unsaturated fatty acids were down-regulated in the aged liver model. In addition, Fgf21 may play a vital role in the transcriptomics-metabolomics co-expression network. As revealed using models of senescent hepatocytes, the expression of FGF21 was also decreased in senescent hepatocytes. Moreover, transcriptomic analysis demonstrated that cell cycle genes were down-regulated significantly and that the down-regulated gene E2F8 was positively related to the cell cycle. We then found that overexpression of the hepatokine FGF21 can protect against liver aging. Furthermore, we found that FGF21 can inhibit the declines in antioxidant and regenerative abilities that occur during liver aging. We also validated our results from cellular and animal experiments using human liver and blood samples and concluded that FGF21 is an important therapeutic target in inhibiting liver aging.

P16, P21, P53, SA-β-Gal, and senescence-associated secretory phenotype markers including Hmgb1, Mmp3, and Lamin B1, are all senescence markers whose levels are increased in the aged liver and hepatocyte models.^49^ The regenerative ability of the aged liver is compromised, and the present study showed that the levels of cyclin A2, B1, D1, E1, and PCNA were decreased in the aged liver model.^3^^,^^45^ We explored the mechanism by multiomics analysis. We performed a transcriptomic assay of aged rat livers and identified 297 down-regulated DEGs and 193 up-regulated DEGs in the intersection of the DEGs identified from the D500 *vs*. D0 and D1000 *vs*. D0 comparisons. These down-regulated DEGs were significantly enriched in the peroxisome and fatty acid metabolism pathways, whereas the up-regulated DEGs were significantly enriched in the steroid biosynthesis and terpenoid backbone biosynthesis pathways. The peroxisome and fatty acid metabolism pathways are both aging-related pathways,^39^^,^^40^ and the significance and importance of these pathways were markedly greater than those of the up-regulated pathways. Weir HJ et al reported that functional coordination of the mitochondrial network with peroxisomes can increase fatty acid oxidation to promote longevity.^39^ Additionally, Pascual-Ahuir A et al reported that the structural and functional connection of mitochondria and peroxisomes is critical to the β-oxidative degradation of fatty acids to protect against aging.^40^ Hence, we performed further studies of peroxisomes and fatty acid metabolism. First, we found 17 TFs among these 297 down-regulated DEGs. We next performed a correlation analysis between these TFs and DEGs in these two down-regulated pathways. These results indicated that Creb3l3 was the key TF correlated with these two pathways. In addition, the levels of the hepatokine Fgf21 were significantly lower in the D500 and D1000 groups than in the D0 group. Fgf21 is significantly correlated with Creb3l3 and is important to longevity.^11^ Gao Y et al found that dietary intervention and exercise-induced Fgf21 expression can regulate AMPK-dependent lipophagy to ameliorate high-fat diet-induced liver aging.^12^ Similarly, Byun S et al found that fasting-induced Fgf21 expression improves defective autophagy and hepatic steatosis in obese mice.^10^^,^^12^ Cristal found that Fgf21 is pivotal in the process by which protein restriction improves metabolic health and extends the lifespan.^11^ Indeed, the circulating levels of Fgf21 are primarily determined by secretion by the liver. Energy expenditure, glucose metabolism, and the thermoregulatory marker UCP1 are increased by Fgf21.^50^ Our RNA-seq results showed that hepatokine Fgf21 may play an essential role in improving peroxisomal function and fatty acid metabolism to protect against liver aging.

The metabolomic assay identified 43 down-regulated-overlapping DEMs and 38 up-regulated-overlapping DEMs in the livers of rats. Forty-three down-regulated DEMs were enriched in the biosynthesis of unsaturated fatty acids (docosahexaenoic acid and arachidonic acid); tyrosine metabolism; and phenylalanine, tyrosine, and tryptophan biosynthesis (phenols and carboxylic acids and derivatives). A diet enriched with unsaturated fatty acids can effectively improve liver fat and lipid metabolism in older people^51^; thus, unsaturated fatty acids are vital for the homeostasis of fat metabolism. The joint-pathway analysis involving the integration of transcriptomics and metabolomics further demonstrated a decline in unsaturated fatty acid biosynthesis. Han Q et al also reported that the levels of carboxylic acids and derivatives from tyrosine metabolism and phenylalanine, tyrosine, and tryptophan biosynthesis are decreased in aged livers.^52^ A pathway analysis of the up-regulated and overlapping DEMs revealed significant enrichment of galactose metabolism (organooxygen compounds), pyruvate metabolism (organooxygen compounds and organic phosphoric acids and derivatives), linoleic acid metabolism (fatty acyls), and primary bile acid biosynthesis (organic sulfonic acids/derivatives and steroids and steroid derivatives). Han Q et al reported that the levels of fatty acyls from linoleic acid metabolism are also significantly decreased in aged rat livers.^52^ Fgf21 was positively correlated with Creb3l3 and plays important roles in the Creb3l3-mediated transcriptomic-metabolomic regulatory network.

We then performed a transcriptomic analysis of the models of senescent hepatocytes. The results showed a decreasing trend in the expression of FGF21 in senescent in contrast with control hepatocytes. Moreover, WB indicated that the expression of hepatokine FGF21 was decreased in aged hepatocytes, which was similar to the results in rats. Some studies have found that FGF21 improves lipid metabolism and glucose metabolism to provide energy for hepatocytes.^53^ Energy expenditure is necessary for hepatic regeneration, and we thus speculate that FGF21 plays a vital role in the process of promoting liver regeneration. An enrichment analysis of DEGs showed that the cell cycle was significantly down-regulated. Livers from old mice cannot activate S-phase-specific genes, and the cell cycle is thus delayed.^45^ Hence, we performed further study of the cell cycle. A Pearson correlation analysis between these TFs and down-regulated DEGs in the cell cycle was conducted, and we found that E2F8 was the most important TF. E2F8 is a gene that participates in the cell cycle and is essential for DNA replication.^45^ A co-expression network between E2F8 and key genes in the cell cycle (MCM3, MCM4, CDK2, BUB1B, CDC25A, SMC1A, CDK1, BUB1, and POLA1) was subsequently constructed. We further demonstrated that E2F8 regulates the expression of these key genes except CDK1. Protein–protein interaction networks indicated that BUB1, CDK1, and BUB1B were more strongly correlated with E2F8, which demonstrated that these genes are important in liver regeneration. The aged liver has a compromised capability for regeneration, which causes a reduced rate of liver regeneration.^54^ Goikoetxea-Usandizaga N et al reported that livers from old donors are more susceptible to ischemia-reperfusion injury than livers from young donors, and mitochondrial dysfunction, reactive oxygen species accumulation, and ATP depletion are major hindrances to liver regeneration.^3^ Overall, the aged hepatocytes exhibit a reduced regeneration ability.

To study the effect of FGF21 on liver aging, FGF21 was overexpressed in the cells and livers of rats. The levels of the senescence markers P16, P21, and P53 in the liver were significantly decreased in the AAV-Fgf21-D1000 group compared with the AAV–NC–D1000 group. The number of SA-β-Gal-positive cells in the AAV-Fgf21-D1000 group was also lower than that in the AAV–NC–D1000 group. Moreover, the levels of the senescence-associated secretory phenotype markers Mmp3 and Lamin B1 were significantly decreased in the AAV-Fgf21-D1000 group. In line with these findings, P16, P21, and P53 were significantly decreased in the HepG2 cells transfected with FGF21-pcDNA compared with the NC-H1000 cells. Similar to the results obtained from rats, the percentage of SA-β-Gal-stained (+) cells was lower among the FGF21-pcDNA transfected cells than among the NC-H1000 cells. SA-β-Gal, a lysosomal enzyme, was the first widely used biomarker for detecting senescent cells.^49^^,^^55^^,^^56^ P16, P21, and p53 are well-known senescence markers,^56^ and the levels of all these markers were decreased in livers and cells overexpressing FGF21. Senescence-associated secretory phenotype markers may self-reinforce senescence or affect the local tissue microenvironment of senescent hepatocytes and possibly the entire liver, supporting a reciprocal influence between aged hepatocytes and senescence-associated secretory phenotypes.^49^ Altogether, the evidence from these experiments in rats and cells supports the notion that FGF21 can protect against liver aging.

Notably, the presence of increased cell cycle-inhibitory proteins, which are cyclin-dependent kinase inhibitors, is a distinctive feature of senescent hepatocytes and causes aged livers to have a reduced regeneration capability.^49^^,^^57^^,^^58^ In our study, the expression of the cell cycle inhibitors P16, P21, and P53 was decreased in the upFGF21-H1000 group.^59^, ^60^, ^61^ Previous studies have reported that increases in the P16, P21, and P53 levels can inhibit the proliferation of cells and promote cellular senescence.^61^^,^^62^ The expression of E2F8 and PCNA was significantly higher in the HepG2 cells of the upFGF21-H1000 group than those of the NC-H1000 group.^63^ In addition, fewer cells in aged livers enter the S phase,^45^ but in our study, the number of cells in the S phase was higher in the upFGF21-H1000 group than in the NC-H1000 group, as determined by flow cytometry. In line with these results, an analysis of the rat model showed that the levels of cyclin D1, cyclin E1, and PCNA were significantly higher in the livers of the AAV-Fgf21-D1000 group than in those of the AAV–NC–D1000 group. Overall, these data demonstrate that FGF21 can protect against not only liver aging but also senescence-associated proliferation arrest.

Aged livers have a decreased antioxidant ability and lower tolerance of oxidative stress, through which reactive oxygen species accumulate in cells, leading to DNA and protein damage.^64^ In line with this finding, the SOD and catalase levels were decreased in aged livers but increased in livers treated with AAV-Fgf21. MDA, an oxidative stress marker, is an end-product of lipid peroxidation with highly reactive properties.^65^^,^^66^ In this study, the liver MDA levels declined in the AAV-Fgf21-D1000 group, and Elham reported that the MDA levels increased in the livers of aged rats.^20^ We then found that the expression of Creb3l3, which is related to peroxisomes and fatty acid metabolism, was increased in the AAV-FGF21-D1000 group. Altogether, these results indicate that FGF21 can not only protect against liver aging but also enhance the oxidative response. Many studies have investigated the role of FGF21 in liver metabolism and have found that FGF21 improves hepatic lipid metabolism and insulin sensitivity to attenuate hepatic steatosis and increase energy expenditure.^67^, ^68^, ^69^ Relatively few studies have focused on the effect of FGF21 on liver aging. In this study, we found that FGF21 improves liver aging and further inhibits the declines in antioxidant and regenerative abilities; these effects have not been reported previously. Moreover, our study provides a new theoretical basis for further research on targeted therapies for liver aging in the future.

We comprehensively introduced the molecular mechanisms of liver aging and hepatocyte senescence from the perspectives of transcriptomics and metabolomics. Moreover, we also preliminarily validated these results using human liver and blood samples. The DEGs and DEMs of aged livers, which were used for enrichment analyses, were obtained by intersecting the DEGs/DEMs obtained from the D500 *vs*. D0 and D1000 *vs*. D0 comparisons. Similarly, the DEGs of senescent hepatocytes, which were used for enrichment analyses, were obtained from the intersection of those obtained from the D40 *vs*. D0, D80 *vs*. D0, H500 *vs*. H0, and H1000 *vs*. H0 comparisons. First, transcriptomic data demonstrated that genes related to peroxisomes and fatty acid metabolism were down-regulated in aged livers. In addition, metabolomic data of aged livers showed that the biosynthesis of unsaturated fatty acids was decreased. In contrast, transcriptomic data from senescent hepatocytes showed that the cell cycle was significantly down-regulated, which revealed that the ability of cells to regenerate declined. Peroxisomes, fatty acid metabolism, and liver regeneration are interconnected.^70^ The decrease in peroxisomes showed that aged livers have a reduced ability to withstand oxidative stress and that lipid metabolism and fatty acid oxidation are thus compromised. Such compromised fatty acid metabolism leads to a decreased energy supply, but liver regeneration is energy-consuming, which decreases the capability for liver regeneration.^71^^,^^72^ Further molecular experiments are needed to further investigate the underlying molecular mechanism.

There are differences in the omics data between hepatocytes and livers from rats, which is a limitation of our study. Indeed, hepatocyte senescence models are induced *in vitro* and thus lack the whole microenvironment of the liver, and thus, these models cannot fully mimic liver aging *in vivo*. Moreover, *in vitro* experiments are relatively simple and different from complex *in vivo* experiments. In addition, the time of induction is only two days in hepatocyte senescence models, but the induction time of the aged liver model is up to 56 days. The aged liver model of rats can reflect liver aging more comprehensively than hepatocyte senescence models. Hence, the interconnection between omics data from rats and omics data from cells is rare. However, the induction method of the *in vitro* hepatocyte senescence model is also scientific. Moreover, the data from the hepatocyte senescence model indicated that the regenerative capacity of the senescent hepatocytes was compromised, which also reflected an aspect of the characteristics of liver aging. Together, omics data from rats and omics data from cells are complementary, and these data reflect the essence and significance of liver aging from different perspectives. In future studies, we can use liver organoids to better mimic natural liver aging *in vitro*.

In conclusion, these results showed that peroxisome and fatty acid metabolism processes decline during liver aging. In addition, senescent hepatocytes have a compromised regenerative ability. Our data showed that overexpressed FGF21 can protect against liver aging and can thereby inhibit senescence-associated decreases in the regenerative and antioxidant capabilities. Hence, FGF21 is an important target for combating the liver aging process. FGF21 can be used to attenuate liver aging-related diseases and reduce the incidence of graft failure after liver transplantation in the future.

## Author contributions

Conception and design: Z Liu, L Zhou, and S Zheng; Administrative support: None; Provision of study materials or patients: None; Collection and assembly of data: Z Liu, W Wang, and M Shang; Data analysis and interpretation: Z Liu and W Wang; Manuscript writing: All authors; Final approval of manuscript: All authors.

## Conflict of interests

The authors have no competing interests to declare.

## Funding

This study was supported by the Research Unit Project of the 10.13039/501100005150Chinese Academy of Medical Sciences (No. 2019-I2M-5-030), the Research Project of Jinan Microecological Biomedicine Shandong Laboratory (China) (No. JNL-2022002A), and the Fundamental Research Funds for the Central Universities (China) (No. 226-2023-00107).

## Data availability

The data used to support the findings of this study are available from the corresponding author upon reasonable request.
