# Supplementary material for: Integrative analysis of the transcriptome and metabolome reveals the importance of hepatokine FGF21 in liver aging
Source: Genes Dis. 2023 Nov 7;11(5):101161. doi: 10.1016/j.gendis.2023.101161 (PMC11252782; doi:10.1016/j.gendis.2023.101161)
Supplement: Multimedia component 1 [file mmc1.docx]

**Bioinformatics analysis for metabolomics**

**Metabolite ion peak extraction and metabolite identification**

We imported the off-line mass spectrometry data into Compound Discoverer 3.2 (Thermo Fisher Scientific, USA, version 3.2) software and analyzed the mass spectrometry data using the Human Metabolome Database (hmdb), the mzCloud database and the ChemSpider online database. We then obtained a data matrix containing information such as the metabolite peak area and identification results. Afterward, we further analyzed and processed the table.

Software information: Compound Discoverer version 3.2; Parameters, (1) parent ion mass deviation < 5 ppm, (2) mass deviation of fragment ions < 10 ppm, (3) retention time deviation < 0.2 min, (4) official website = https://mycompounddiscoverer.com/.

**2. Data preprocessing**

The resulting file obtained from Compound Discoverer was inputted into MetaX for data preprocessing and further analysis[1]. The data preprocessing included the following: (1) probabilistic quotient normalization (PQN) was used to normalize the data to obtain relative peak areas[2]. ;(2) we corrected the batch effect by quality control-based robust LOESS signal correction (QC-RLSC) **[3]**; and (3) metabolites with a relative peak area with a coefficient of variation greater than 30% were removed from the QC samples.

**3. Annotation of metabolites and pathway enrichment analysis (KEGG)**

The taxonomic and functional annotation of the identified metabolites is helpful in understanding the properties of different metabolites. The hmdb includes chemical, molecular biology/biochemical, and clinical information on metabolites to support metabolic pathways and spectral searches. The Kyoto Encyclopedia of Genes and Genomes (KEGG) pathways are the core of the KEGG database and include many metabolic pathways and the relationships among them. In organisms, different metabolites correspond to each other to perform their biological functions. The KEGG pathway database was used for functional annotation of pathways to identify the main biochemical metabolic pathways that involve the metabolites.

**4. Screening of the differences between groups**

Partial least squares-discriminant analysis (PLS-DA) is a supervised statistical method that can better reflect the differences among classification groups. In PLS-DA, partial least squares regression was used to build a model between metabolite expression and sample categories to predict sample categories. Additionally, the variable importance for the projection (VIP) was used to measure the impact strength and explanatory power of each metabolite expression pattern on the classification and discrimination of each group of samples and help identify metabolic biomarkers. In general, a VIP value > 1 demonstrates that the corresponding metabolite has significance in distinguishing the sample categories. After the log2 transformation of the data, a PLS-DA model was established among the compared groups, Pareto was used as the scaling method, and 7-fold cross-validation was used to validate the constructed model. OPLS-DA, which is a combination of OSC and PLS-DA, is an extension of PLS-DA and can decompose X matrix information into two types of information related to Y and unrelated to Y, remove information irrelevant to classification, and effectively reduce the complexity of the model without reducing its predictive ability, enhancing the explanatory ability of the model.

**Bioinformatics analysis of transcriptomics**

**1. Sequence and filtering of clean reads**

A cDNA library constructed from the pooled RNA from the liver tissues of rats and HepG2 cells was sequenced with the Illumina NovaSeqTM 6000 sequence platform. We sequenced the transcriptome, and a total of 2 x 150-bp paired-end reads were generated using the Illumina paired-end RNA-seq approach. Reads acquired from the sequencing machines contain raw reads with adapters or low-quality bases, which will affect the subsequent assembly and analysis. Hence, Cutadapt (https://cutadapt.readthedocs.io/en/stable/, version: 1.9) was used to further filter reads to obtain high-quality clean reads. The parameters are listed as follows:

1) reads with adapters were removed;

2) reads with polyA and polyG were removed;

3) reads including more than 5% of unknown nucleotides (N) were removed; and

4) low-quality reads with more than 20% low-quality (Q-value≤20) bases were removed.

FastQC was used to validate the sequence quality containing the Q20, Q30, and GC content of the clean data. Afterward, we obtained G bp of cleaned, paired-end reads [4].

**2. Alignment with the reference genome**

We used the HISAT2 (https://daehwankimlab.github.io/hisat2/, version: 2.0.4) package to align reads from all the samples to the rat and human reference genomes, which initially involved removing a portion of the reads based on quality information for each read followed by mapping the reads to the reference genome[5, 6]. When reads are mapped to the reference, HISAT2 allows a maximum of two mismatches and multiple alignments per read (up to 20 by default). HISAT2 builds a database of potential splice junctions and confirms these by comparing the previously unmapped reads against the database of putative junctions[7].

**3. Quantification of gene abundance**

StringTie with the default parameters was used to assemble the mapped reads of every sample. GffCompare software (http://ccb.jhu.edu/software/stringtie/gffcompare.shtml, version: 0.9.8) was then used to reconstruct a comprehensive transcriptome by merging all transcriptomic data from all the samples. We then estimated the expression levels of all transcripts and determined the expression abundance of mRNAs by calculating FPKM (fragment per kilobase of transcript per million mapped reads) values using StringTie and Ballgown (<http://www.bioconductor.org/packages/release/bioc/html/ballgown.html>)[6, 8, 9].

**4. Analysis of relationships among samples**

**4.1. Correlation analysis of replicates**

R was used for correlation analysis, and the reliability of the experimental results and operational stability were evaluated by the correlations among two parallel experiments. We calculated the Pearson correlation coefficient between two replicates to evaluate the repeatability among samples. The closer the correlation coefficient is to 1, the better the repeatability of the two parallel experiments.

**4.2. Principal component analysis**

PCA can convert hundreds of thousands of correlated variables (gene expression) into a set of values of linearly uncorrelated variables called principal components and reveals the structure/relationship of the samples/data.

**4.3. Pathway enrichment analysis (KEGG)**

Genes play important roles in certain biological functions by interacting with each other. The biological functions of genes can be further understood by pathway-based analysis. KEGG is the major public pathway-related database(https://www.kegg.jp/kegg/). Significantly enriched metabolic pathways or signal transduction pathways were identified by a pathway enrichment analysis of DEGs compared with the whole genome background [10]. The formula for calculating the P value is the following:

In this formula, N is the number of all genes with KEGG annotation, n is the number of DEGs in N, M is the number of all genes annotated to specific pathways, and m is the number of DEGs in M. Pathways with P < 0.05 were considered significantly enriched pathways.

**Table S3** Information of software packages used for transcriptome and metabolome analysis.

| **Software**  **Name** | **Version Number** | **Reference** |
| --- | --- | --- |
| HISAT2 | 2.0.4 | HISAT: a fast spliced aligner with low memory requirements. |
| DESeq2 | 1.40.2 | Moderated estimation of fold change and dispersion for RNA-seq data with DESeq2. |
| Pathview | 1.40.0 | Pathview: an R/Bioconductor package for pathway-based data integration and visualization. |
| Cytoscape | 3.9.1 | Cytoscape: A Software Environment for Integrated Models of Biomolecular Interaction Networks. |
| CytoHubba | 0.1 | cytoHubba: identifying hub objects and sub-networks from complex interactome. |
| STRING | 12.0 | STRING v11: protein-protein association networks with increased coverage, supporting functional discovery in genome-wide experimental datasets. |
| Compound Discoverer | 3.2 | Compound Discoverer as a tool for bioprospecting and detection of defense compounds during fungal infection of Spruce wood. |
| MetaX | 1.0 | MetaX a flexible and comprehensive software for processing metabolomics data. |
| Cutadapt | 1.9 | Cutadapt Removes Adapter Sequences From High-Throughput Sequencing Reads. |
| FastQC | 0.11.9 | FastQC: A Quality Control Tool for High Throughput Sequence Data [Online]. Available online at: <http://www.bioinformatics>.babraham.ac.uk/projects/fastqc/. |
| StringTie | 2.2.0 | StringTie enables improved reconstruction of a transcriptome from RNA-seq reads. |
| GffCompare | 0.12.6 | GFF Utilities: GffRead and GffCompare. |
| Ballgown | 2.32.0 | Transcript-level expression analysis of RNA-seq experiments with HISAT, StringTie and Ballgown. |

**Reference:**

1. Wen B, Mei Z, Zeng C, Liu S: **metaX: a flexible and comprehensive software for processing metabolomics data**. *BMC bioinformatics* 2017, **18**(1):183.

2. Di Guida R, Engel J, Allwood JW, Weber RJM, Jones MR, Sommer U, Viant MR, Dunn WB: **Non-targeted UHPLC-MS metabolomic data processing methods: a comparative investigation of normalisation, missing value imputation, transformation and scaling**. *Metabolomics* 2016, **12**:93.

3. Dunn WB, Broadhurst D, Begley P, Zelena E, Francis-McIntyre S, Anderson N, Brown M, Knowles JD, Halsall A, Haselden JN *et al*: **Procedures for large-scale metabolic profiling of serum and plasma using gas chromatography and liquid chromatography coupled to mass spectrometry**. *Nat Protoc* 2011, **6**(7):1060-1083.

4. Thompson O, von Meyenn F, Hewitt Z, Alexander J, Wood A, Weightman R, Gregory S, Krueger F, Andrews S, Barbaric I *et al*: **Low rates of mutation in clinical grade human pluripotent stem cells under different culture conditions**. *Nat Commun* 2020, **11**(1):1528.

5. Kim D, Paggi JM, Park C, Bennett C, Salzberg SL: **Graph-based genome alignment and genotyping with HISAT2 and HISAT-genotype**. *Nat Biotechnol* 2019, **37**(8):907-915.

6. Pertea M, Kim D, Pertea GM, Leek JT, Salzberg SL: **Transcript-level expression analysis of RNA-seq experiments with HISAT, StringTie and Ballgown**. *Nat Protoc* 2016, **11**(9):1650-1667.

7. Kim D, Langmead B, Salzberg SL: **HISAT: a fast spliced aligner with low memory requirements**. *Nat Methods* 2015, **12**(4):357-360.

8. Kovaka S, Zimin AV, Pertea GM, Razaghi R, Salzberg SL, Pertea M: **Transcriptome assembly from long-read RNA-seq alignments with StringTie2**. *Genome Biol* 2019, **20**(1):278.

9. Pertea M, Pertea GM, Antonescu CM, Chang T-C, Mendell JT, Salzberg SL: **StringTie enables improved reconstruction of a transcriptome from RNA-seq reads**. *Nat Biotechnol* 2015, **33**(3):290-295.

10. Kanehisa M, Furumichi M, Sato Y, Ishiguro-Watanabe M, Tanabe M: **KEGG: integrating viruses and cellular organisms**. *Nucleic Acids Res* 2021, **49**(D1):D545-D551.
