# Supplementary material for: Integrative analysis of the transcriptome and metabolome reveals the importance of hepatokine FGF21 in liver aging
Source: Genes Dis. 2023 Nov 7;11(5):101161. doi: 10.1016/j.gendis.2023.101161 (PMC11252782; doi:10.1016/j.gendis.2023.101161)
Supplement: Multimedia component 2 [file mmc2.docx]

**Table S1** Primer sequences for the target genes.

| **Species** | **Target** | **Sequence of RNA** |
| --- | --- | --- |
| Rat | Fgf21-Forward | CCTTGAAGCCAGGGGTCATT |
| Rat | Fgf21-Reverse | GGATCAAAGTGAGGCGATCC |
| Rat | P16-Forward | GTAGTACTGCACCAGGCAGG |
| Rat | P16-Reverse | CCCAGCGGAGGAGAGTAGAT |
| Rat | P21-Forward | ATCGAGACACTCAGAGCCACA |
| Rat | P21-Reverse | CGTCTCAGTGGCGAAGTCAA |
| Rat | P53-Forward | CCCCTGAAGACTGGATAACTGT |
| Rat | P53-Reverse | AATTAGGTGACCCTGTCGCTG |
| Rat | GAPDH-Forward | CCGCATCTTCTTGTGCAGTG |
| Rat | GAPDH-Reverse | CGATACGGCCAAATCCGTTC |
| Homo | FGF21-Forward | CTGTGGGTTTCTGTGCTGG |
| Homo | FGF21-Reverse | CCGGCTTCAAGGCTTTCAG |
| Homo | P16-Forward | CTCGTGCTGATGCTACTGAGGA |
| Homo | P16-Reverse | GGTCGGCGCAGTTGGGCTCC |
| Homo | P21-Forward | AGGTGGACCTGGAGACTCTCAG |
| Homo | P21-Reverse | TCCTCTTGGAGAAGATCAGCCG |
| Homo | P53-Forward | CCTCAGCATCTTATCCGAGTGG |
| Homo | P53-Reverse | TGGATGGTGGTACAGTCAGAGC |
| Homo | GAPDH-Forward | GTCTCCTCTGACTTCAACAGCG |
| Homo | GAPDH-Reverse | ACCACCCTGTTGCTGTAGCCAA |
