# Supplementary material for: Integrative analysis of the transcriptome and metabolome reveals the importance of hepatokine FGF21 in liver aging
Source: Genes Dis. 2023 Nov 7;11(5):101161. doi: 10.1016/j.gendis.2023.101161 (PMC11252782; doi:10.1016/j.gendis.2023.101161)
Supplement: Multimedia component 3 [file mmc3.docx]

**Table S2** Quantity analysis of immunohistochemical staining.

| **Score of positive cells percentage (SPCP)** | **Score of staining intensity (SSI)** | **Score of relative expression** |
| --- | --- | --- |
| 0 = no PPs | 0 = no color reaction | SPCP × SSI |
| 1 = ≤25% PPs | 1 = mild reaction |  |
| 2 = 25% - 50% PPs | 2 = moderate reaction |  |
| 3 = 50% - 75% PPs | 3 = intense reaction |  |
| 4 = > 75% PPs |  |  |

SPCP, score of positive cells percentage; SSI, score of staining intensity.
